# Evolutionary conserved relocation of chromatin remodeling complexes to the mitotic apparatus

**DOI:** 10.1186/s12915-022-01365-5

**Published:** 2022-08-03

**Authors:** Giovanni Messina, Yuri Prozzillo, Francesca Delle Monache, Maria Virginia Santopietro, Patrizio Dimitri

**Affiliations:** 1 Dipartimento di Biologia e Biotecnologie “Charles Darwin”, Sapienza Università di Roma, Rome, Italy; 2 Istituto Pasteur Italia, Fondazione Cenci Bolognetti, Rome, Italy

**Keywords:** Chromatin remodeling, Moonlighting proteins, Cell division, Cytokinesis, Midbody

## Abstract

**Background:**

ATP-dependent chromatin remodeling complexes are multi-protein machines highly conserved across eukaryotic genomes. They control sliding and displacing of the nucleosomes, modulating histone-DNA interactions and making nucleosomal DNA more accessible to specific binding proteins during replication, transcription, and DNA repair, which are processes involved in cell division. The SRCAP and p400/Tip60 chromatin remodeling complexes in humans and the related *Drosophila* Tip60 complex belong to the evolutionary conserved INO80 family, whose main function is promoting the exchange of canonical histone H2A with the histone variant H2A in different eukaryotic species. Some subunits of these complexes were additionally shown to relocate to the mitotic apparatus and proposed to play direct roles in cell division in human cells. However, whether this phenomenon reflects a more general function of remodeling complex components and its evolutionary conservation remains unexplored.

**Results:**

We have combined cell biology, reverse genetics, and biochemical approaches to study the subcellular distribution of a number of subunits belonging to the SRCAP and p400/Tip60 complexes and assess their involvement during cell division progression in HeLa cells. Interestingly, beyond their canonical chromatin localization, the subunits under investigation accumulate at different sites of the mitotic apparatus (centrosomes, spindle, and midbody), with their depletion yielding an array of aberrant outcomes of mitosis and cytokinesis, thus causing genomic instability. Importantly, this behavior was conserved by the *Drosophila melanogaster* orthologs tested, despite the evolutionary divergence between fly and humans has been estimated at approximately 780 million years ago.

**Conclusions:**

Overall, our results support the existence of evolutionarily conserved diverse roles of chromatin remodeling complexes, whereby subunits of the SRCAP and p400/Tip60 complexes relocate from the interphase chromatin to the mitotic apparatus, playing moonlighting functions required for proper execution of cell division.

**Supplementary Information:**

The online version contains supplementary material available at 10.1186/s12915-022-01365-5.

## Background

Cell division is a fundamental event common to most life forms. In eukaryotes, successful cell division requires the balanced distribution of chromosomes and cytoplasmic material to daughter cells. This is achieved via a series of coordinated cytoskeletal processes which include centrosome-mediated spindle assembly, spindle positioning, chromosome segregation, and cytokinesis [1, 2]. Once chromosome segregation is complete, the actomyosin contractile ring is assembled at the cleavage furrow and drives the constriction of the plasma membrane where abscission, the final stage of cytokinesis, will occur. Before abscission, the two newly generated daughter cells are still connected by a cytoplasmic bridge that contains the midbody, a mitotic structure first described by Walther Flemming at the end of the 1800s [3].

**Fig. 1:**
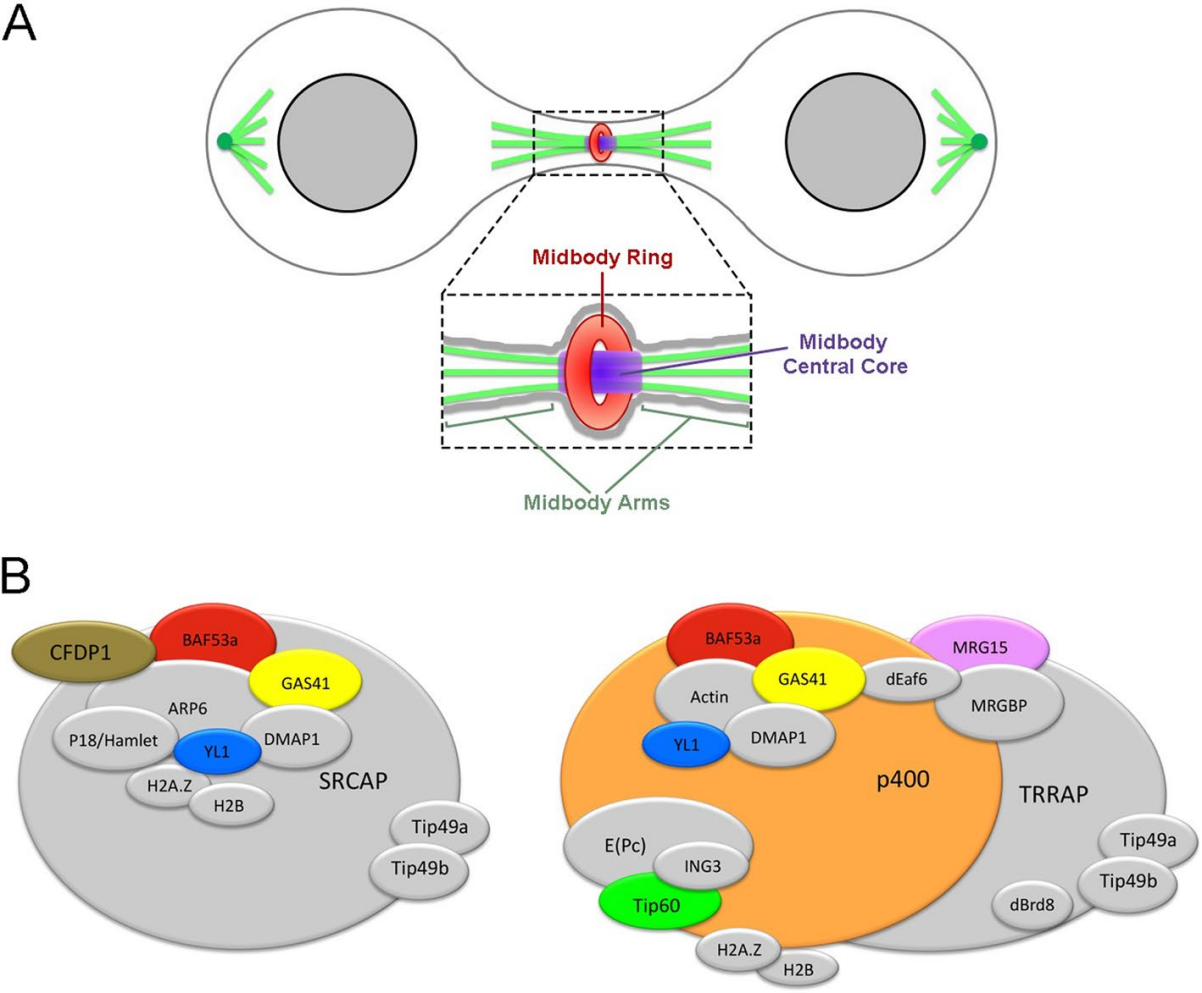
Schematic representation of midbody and subunit composition of SRCAP and p400/Tip60 chromatin remodeling complexes in human cells. **A** The midbody can be subdivided in three main regions [8, 35]: the midbody ring (red), containing anillin and citron kinase and other contractile ring components; the midbody central core (blue) marked by central spindle proteins such as the centralspindlin complex; and the midbody arms (green) where Aurora B and the other components of the chromosomal passenger complex (CPC) are recruited. **B** SRCAP (left) and p400/Tip60 (right) complexes share many subunits. The chromatin remodeling subunits (CRS) studied in this work are depicted in colours

The midbody has become the focus of intense investigation in cell biology. It is a tightly packed structure that forms from the bipolar microtubule array of central spindle and plays essential functions in localizing the site of abscission, and hence of physical separation of daughter cells during cytokinesis (Fig. 1A [4–9]). Cytokinesis requires a complex interplay among a plethora of regulatory and effector components related to cytoskeleton, chromosomes, cell cycle, lipid raft, vesicle, and membrane trafficking factors which map to the midbody [4–8, 10–17]. Notably, most of the main actors and mechanisms regulating the different steps of cell division are evolutionary conserved in eukaryotes [4–7, 10–15].

Studies on cell division are increasingly relevant to cancer research. Indeed, chromosome segregation defects during mitosis produce aneuploidy [18, 19] which lead to chromosomal instability, a principal contributor to genetic heterogeneity in cancer and an important determinant of clinical prognosis and therapeutic resistance. Moreover, midbody alterations cause cytokinesis failure [7, 20] and result in (1) inhibition or regression of the cleavage furrow, with the ensuing formation of binucleated cells, or (2) persistence of connections between daughter cells, with the formation of long intercellular bridges originating syncytial cells. Cytokinesis failures results in tetraploid and polyploid cells with multiple centrosomes, which can lead to aneuploid daughter cells that would promote tumorigenic transformation [7, 20–22]. The elucidation of molecular mechanisms underlying mitosis and cytokinesis can therefore contribute to both cancer prognosis and therapy.

A role in cell division was proposed for chromatin remodelers. Pontin, Reptin, and Tip60, subunits of SRCAP (Snf-2-related CREB-binding protein activator protein) p400/Tip60 chromatin remodeling complexes [23], as well as the INO80 remodeler, were shown to relocate to mitotic apparatus and suggested to play direct roles during cell division in human cells [24–29]. However, little is known about relevance and evolutionary conservation of this phenomenon. We have recently found that the ATPase SRCAP, the main subunit of the SRCAP complex, which is implicated in the Floating Harbor syndrome [30], associates with components of the mitotic apparatus (centrosomes, spindle, midbody), interacts with a plethora of cytokinesis regulators, and positively controls their recruitment to the midbody [31]. Remarkably, SRCAP depletion perturbs both mitosis and cytokinesis. Similarly, DOM-A, the functional SRCAP orthologue of *Drosophila melanogaster*, is found at centrosomes and the midbody in this model organism and its depletion affects cell division [31]. Together our findings suggested that SRCAP ensures proper chromosome segregation during mitosis and midbody function during cytokinesis.

In the present work, we have combined cell biology, functional genomics, and biochemical approaches to perform an in-depth investigation in order to (1) analyze the subcellular localization of subunits of SRCAP and p400/Tip60 chromatin remodeling complexes during cell cycle progression in human cells and (2) test their involvement in cell division. The study has been further extended to MRG15, YETI, and Tip60, three subunits of *Drosophila melanogaster* Tip60 complex (dTip60), which are orthologous to those of SRCAP and p400/Tip60 complexes tested (Fig. 1B). The experiments were performed in HeLa cells and *Drosophila* S2 cells, which are powerful systems to study cell division and were used in extensive genome-wide screens to identify mitotic genes [4, 7, 20, 32].

Overall, our results revealed that the subunits of the human SRCAP and p400/Tip60 complexes under investigation, as well as their *Drosophila melanogaster* orthologues, function beyond chromatin remodeling, in that they are massively recruited to the mitotic apparatus and participate to the control of spindle and midbody function during cell division.

## Results

### The subunits of SRCAP and p400/Tip60 chromatin remodeling complexes localize to the mitotic apparatus

Using immunofluorescence microscopy (IFM), we investigated the subcellular localization of seven endogenous subunits of the SRCAP and p400/Tip60 chromatin remodeling complexes during cell division in fixed HeLa cells: CFDP1 (SRCAP complex), MRG15, p400 and Tip60 (p400/Tip60 complex), BAF53a, GAS41, and YL1 (both complexes).

**Fig. 2:**
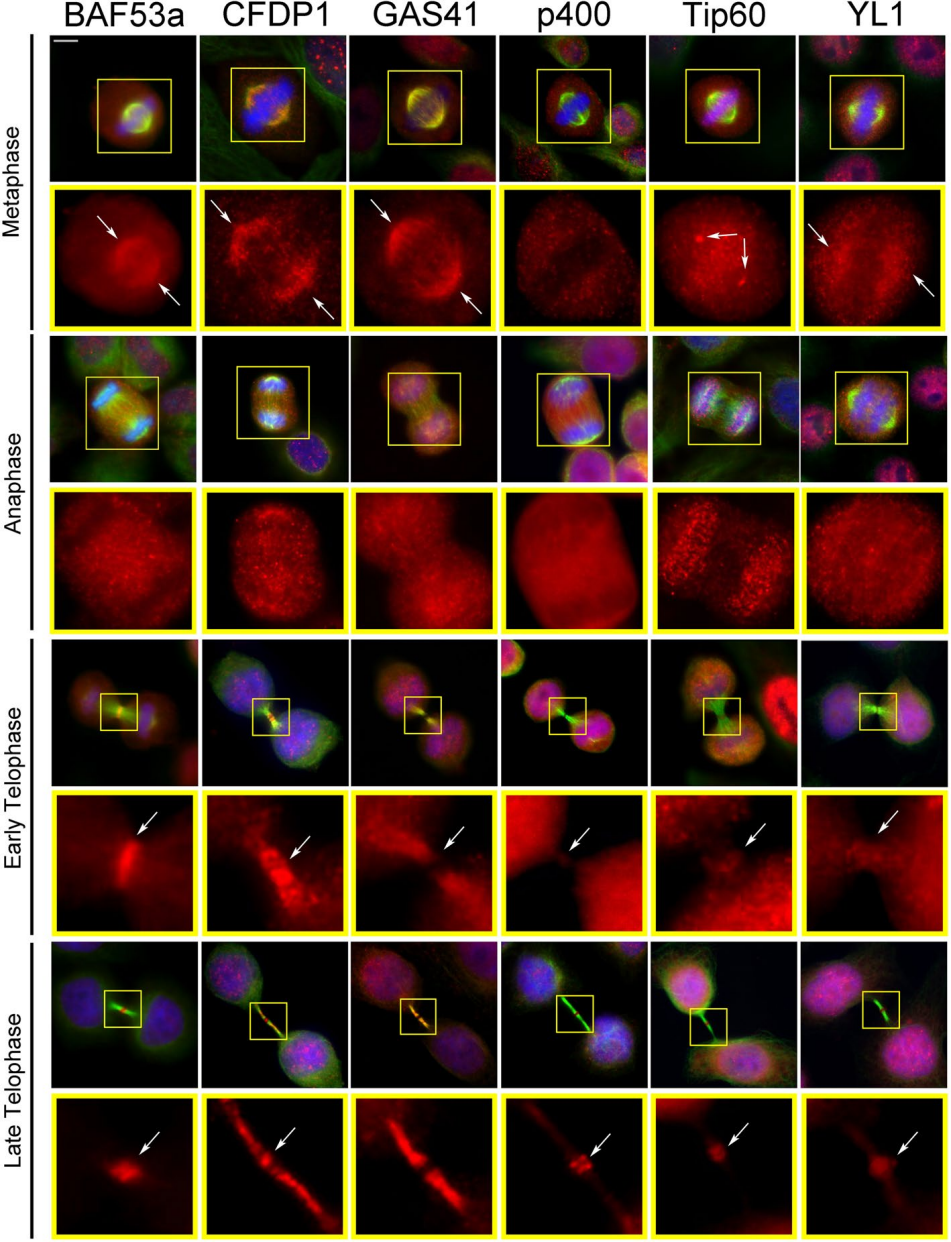
Localization of SRCAP and p400/Tip60 chromatin remodeling subunits (CRS) to the centrosomes, spindle, and midbody in HeLa cells. Fixed HeLa cells stained with DAPI (blue), anti-CRS (red), and anti-α-tubulin (green). BAF53a, CFDP1, GAS41, and YL1 fluorescent signals were found at the spindle in both metaphase and/or anaphase; Tip60 localized at the centrosome and kinetochores (particularly evident at anaphase). In telophase, all the tested CRS were found at the midbody. Scale bar = 10 μm

As expected, for each chromatin remodeling subunit (CRS) tested, the antibody staining decorated the interphase nuclei (Additional file 1: Fig. S1). In addition, we observed a specific staining pattern at the mitotic apparatus (centrosomes, spindle, and midbody) during mitotic progression, with the exception of MRG15 that failed to show accumulation to the mitotic structures (Fig. 2). In particular, BAF53a, CFDP1, GAS41, and YL1 were found at the spindle in both metaphase and/or anaphase; Tip60 localized at the centrosomes and also showed a kinetochore accumulation, as previously reported [33]. Finally, all these subunits were also found to the midbody (Fig. 2 and Table 1).

**Table 1:** Localization of CRS to the mitotic apparatus

CRS	Localization	Reference
BAF53a	Mitotic spindle, cleavage furrow, central spindle, midbody core and ring	This work
CFDP1	Mitotic spindle, central spindle, midbody core and arms	This work
GAS41	Mitotic spindle, central spindle, midbody arms	This work
H2A.Z	Midbody arms	This work
p400	Midbody core	This work
Pontin	Spindle poles, central spindle, midbody arms	This work - Gartner, et al. 2003; Sigala, et al. 2005; Ducat, et al. 2008; Gentili, et al. 2015
Reptin	Midbody core	This work - Sigala, et al. 2005; Gentili, et al. 2015
Tip60	Spindle poles, kinetochores, cleavage furrow, central spindle, midbody core and ring	Zhang, et al. 2012
YL1	Mitotic spindle, central spindle, midbody core	This work

**Fig. 3:**
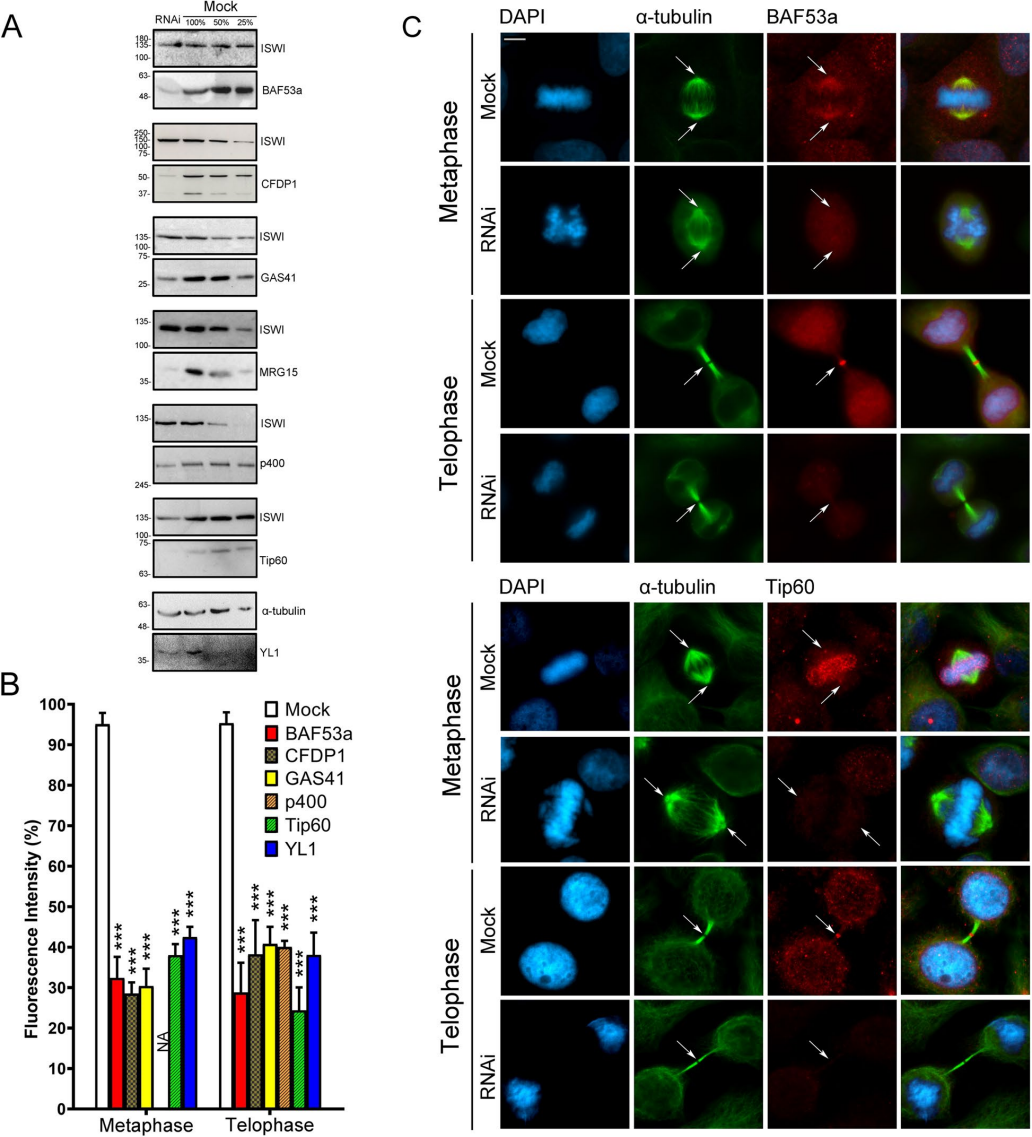
Validation of antibodies against the CRS. **A** Total CRS amount assessed by Western blotting in RNAi-treated and mock-treated cells. **B** Histograms showing the fluorescence intensity of immunostaining in RNAi-treated and mock-treated cells. The colors of bars are referred to Fig. 1B. Bars with different dashed lines indicate SRCAP complex-specific subunits (CFDP1) or p400/Tip60-specific subunits (p400 and Tip60). Plain colored bars indicate subunits common to both complexes (BAF53a, GAS41, and YL1). The fluorescence intensity showed a decrease ranging from about 60 up to 75% in RNAi-treated HeLa cells compared to the mock-treated cells. Fluorescence intensity was assessed using the ImageJ software and statistical significance was verified by *T*- test. **C** Examples of IFM staining. The fluorescence intensity of BAF53a and Tip60 clearly decreased in RNAi-treated compared to mock-treated cells. Scale bar = 10 μm

The antibodies against the CRS of interest were validated by both IFM and Western blotting (WB) on HeLa cells transfected with a specific siRNA mix targeting the transcripts of each gene encoding the CRS (see “Methods”). After RNAi knockdown, the total amount of each CRS decreased, as well as the fluorescence intensity of nuclei, spindles, and midbodies (Fig. 3, Additional file 2: Fig. S2).

The subcellular localizations of the endogenous CRS found in HeLa cells, were also examined in human MRC5 fibroblast-derived cell line. Here again, in addition to chromatin, the CRS accumulated at different sites of the mitotic apparatus (Additional file 3: Fig. S3). These results suggested that the mitotic localizations of the CRS reflect intrinsic properties of the proteins, independently of the cell type.

**Fig. 4:**
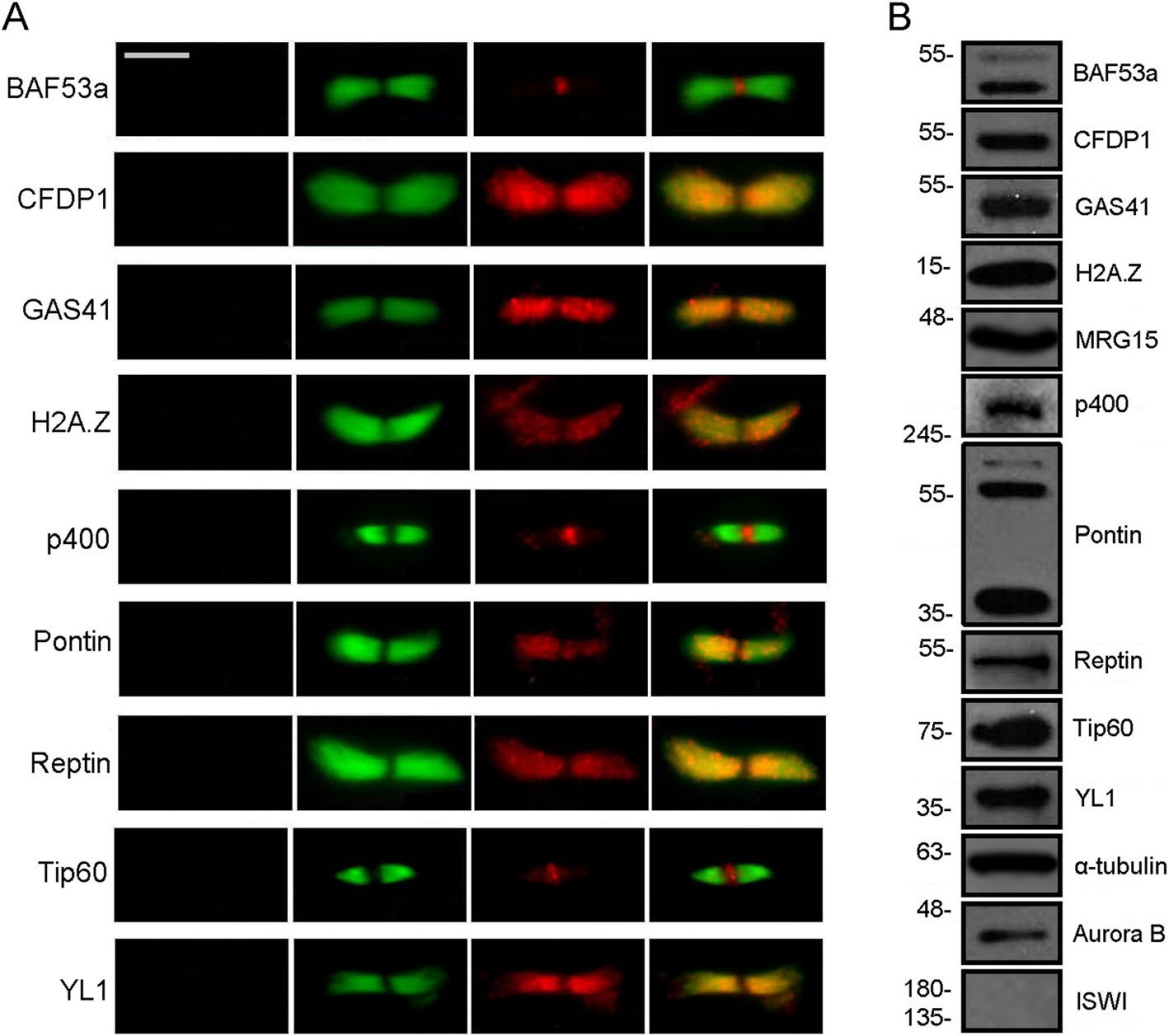
Localization of CRS on isolated midbodies. **A** Immunolocalization. Fixed preparations of isolated midbodies were stained with DAPI (blue), antibodies against the protein of interest (red), and anti-α-tubulin (green). No DAPI staining was detected. The antibody staining for each tested protein clearly decorated the isolated midbodies. Scale bar = 5 μm. **B** Detection of CRS by Western blotting on midbody extracts from HeLa cells. Aurora B and α-tubulin were used as positive controls. ISWI was not detected (negative control)

The midbody associations of CRS were also evaluated on isolated midbodies from HeLa cells using IFM and WB. In addition to the CRS analysed in fixed HeLa cells (Fig. 2), we tested Pontin and Reptin, which were found to localize to the spindle and midbody [24–26, 29]. We also tested the histone variant H2A.Z, which replaces the canonical histone H2A, due to the action of SRCAP and p400/Tip60 complexes [23]. As shown in Fig. 4, all the proteins under investigation were found to the isolated midbodies by both IFM and WB, with the exception of MRG15 that was detected only by WB. By contrast, the ISWI remodeler [34] was not found. Interestingly, the localization of Tip60 at the isolated midbodies was widely distributed in early telophase and became gradually more restricted in late telophase, similarly to that of other known cytokinesis regulators [35].

Taken together, these findings revealed that the subcellular localization of CRS tested is dynamic during cell division, in that they relocate to different structures of the mitotic apparatus. Remarkably, SRCAP and p400/Tip60 complexes belong to the INO80 family of ATP-dependent chromatin remodelers, whose main function is to govern the deposition of the variant histone H2A in evolutionary distant species [23], thus such a massive relocation to the mitotic apparatus was not obvious.

### RNAi-mediated depletion of SRCAP and p400/Tip60 complex subunits affects mitosis and cytokinesis in HeLa cells

Next, we examined the functional relevance of the CRS relocation to the mitotic apparatus, by investigating the progression of cell division after their RNAi-mediated depletion. These experiments were performed by transfecting HeLa cells with specific siRNAs against the transcripts encoding the following CRS: BAF53a, CFDP1, GAS41, MRG15, p400, Tip60, and YL1. Two negative control samples were considered: scramble siRNAs transfected Hela cells and mocktreated HeLa cells (see “Methods”).

**Fig. 5:**
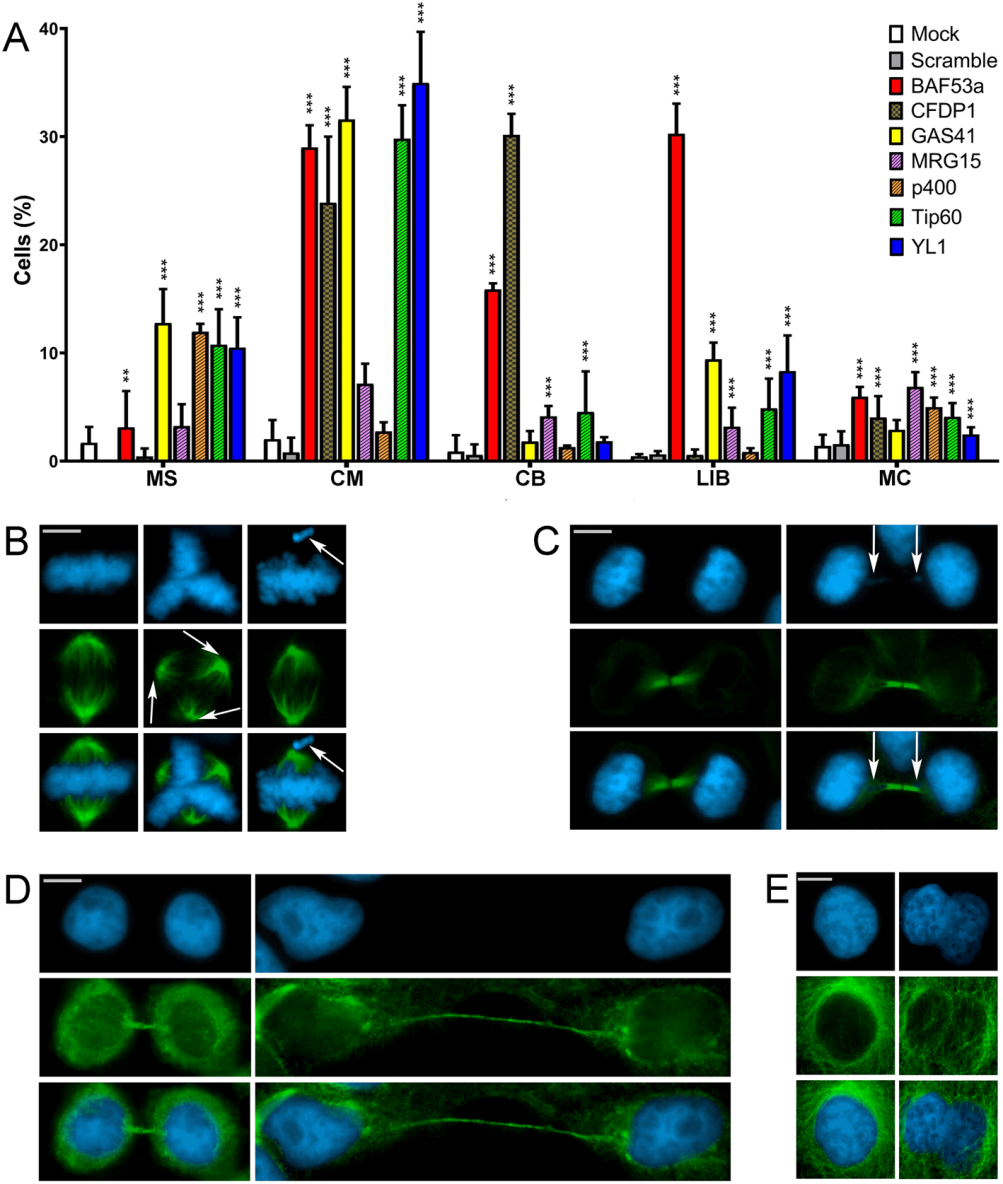
RNAi mediated depletion of CRS affects cell division in HeLa cells. Cells were stained with DAPI (blue) and anti-α-tubulin (green). Scale bar = 10 μm. Five classes of defects were categorized. **A** Histograms showing the quantitative analysis of cell division defects. Colors are referred to Fig. 1B. Bars with two different dashed lines indicate SCRAP complex-specific subunits (CFDP1) or p400/Tip60-specific subunits (MRG15, p400, and Tip60). Plain colored bars indicate subunits common to both complexes (BAF53a, GAS41, and YL1). **B** Mock, left panel; multipolar spindles (MS), middle panel; chromosome misalignments (CM), right panel. **C** Mock, left panel; chromatin bridges (CB) right panel. **D** Mock, left panel; long intercellular bridges (LIB) right panel; no DAPI-stained trapped chromatin was observed. **E** Mock, left panel; multinucleated cells (MC), right panel. Three independent experiments were performed. The quantitative analysis of defects scored in RNAi-treated and control cells (Table 2) is based on the following numbers: at least 100 prometaphases and metaphases for MS, 70 metaphases for CM and ASM, 300 telophases for LIB and CB, and 5500 interphases for MC. Experimental groups were compared with control groups (mocks and scrambles) by Fisher’s exact test.*=*P* < 0.05; **=*P* < 0.005; and ***=*P* < 0.0005

As shown in Fig. 5A–E and Table 2, depletion of each CRS tested gave rise to cells showing five major classes of cell division defects: multipolar spindles (MS), chromosome misalignments in metaphase (CM), chromatin bridges (CB), long intercellular bridges (LIB), and multinucleated cells (MC). The strength of the observed defects varies between CRS, but a clear increase of mitotic abnormalities was observed which in most cases is statistically significant compared to the control samples.

**Table 2:** Cell division defects found in CRS-depleted HeLa cells

		Mock	Scramble	BAF53a	CFDP1	GAS41	MRG15	p400	Tip60	YL1
Metaphase	MS	1.43 ± 1.47	0 ± 0	3.12 ± 3.35	0.43 ± 0.74	12.77 ± 3.13***	3.23 ± 2.01	11.95 ± 0.74***	10.77 ± 3.27***	10.49 ± 2.80***
	CM	2.03 ± 1.76	0.79 ± 1.37	29.00 ± 2.06***	23.90 ± 6.11***	31.60 ± 3.00***	7.16 ± 1.85*^(Scramble)^	2.74 ± 0.85	29.80 ± 3.10***	34.98 ± 4.72***
Telophase	CB	0.87 ± 1.52	0.56 ± 0.98	15.86 ± 0.57***	30.16 ± 1.96***	1.80 ± 0.97	4.13 ± 0.95***	1.29 ± 0.14	4.55 ± 3.75***	1.83 ± 0.39
	LIB	0.44 ± 0.20	0.62 ± 0.30	30.27 ± 2.79***	0.57 ± 0.51	9.41 ± 1.54***	3.19 ± 1.74***	0.84 ± 0.36	4.88 ± 2.75***	8.32 ± 3.29***
	MC	1.40 ± 1.03	1.55 ± 1.21	5.96 ± 0.90***	4.02 ± 1.99***	2.89 ± 0.90***	6.87 ± 1.36***	4.98 ± 0.89***	3.09 ± 1.27***	2.47 ± 0.65***

*MS* multipolar spindles, *CM* chromosome misalignments, *CB* chromatin bridges, *LIB* long intercellular bridges, *MC* multinucleated cells

The results are expressed as mean ± SD values from three independent replicate experiments. Experimental groups were compared with control groups (mocks and scrambles) by Fisher’s exact test. *=*P* < 0.05; **P= < 0.005; and ***=*P* < 0.0005

*^(Scramble)^=This means that it is statistically significant only with the scramble

Depletion of GAS41, p400, Tip60, and YL1 resulted in a significant increase of metaphases showing multipolar spindles (Fig. 5A, B): 12.77, 11.95, 10.77, and 10.49%, respectively, compared to mock and scramble controls. CFDP1 depletion did not result in an increase of multipolar spindle, but led to 32% of metaphases with monopolar spindles (Additional file 4: Fig. S4).

A significant increase of metaphases showing CM (Fig. 5A, B) was detected in BAF53a, CFDP1, GAS41, MRG15, Tip60, and YL1 depleted cells: 29, 23.9, 31.6, 7.16, 29.8, and 35% respectively. A high percentage of telophases with CB (Fig. 5A, C) was found only in BAF53a and CFDP1 depleted cells, while milder effects were seen in MRG15 and Tip60 depleted cells, and no significant effects were observed for the other subunits tested. Notably, mitotic defects in terms of lagging chromosomes were seen after depletion of Pontin [29].

Cytokinesis was also perturbed by depletion of all CRS under investigation. Reduction of BAF53a, GAS41, MRG15, TIP60, and YL1 amounts clearly resulted in dysfunctional abscission, leading to a significant increase in the percentage of figures showing LIB: 30.27, 9.41, 3.19, 4.88, and 8.32% respectively (Fig. 5A, D). Consistently, after depletion of the aforementioned CRS, the intercellular distance during cytokinesis increased (Additional file 5: Fig. S5). Finally, the depletion of all the subunits tested, with the exception of GAS41, also produced a statistically significant increase of MC (Fig. 5A, E).

In summary, a significant increase of mitosis and cytokinesis defects was observed after depletion of SRCAP and p400/Tip60 complex subunits, suggesting that their relocation to mitotic apparatus reflects functional roles in cell division.

### BAF53a andTip60 interact with each other and with cytokinesis regulators in telophase synchronized cells

In a previous report, we have found that the ATPase SRCAP interacts with a plethora of cytokinesis regulators and positively controls their recruitment to the midbody [31].

To further investigate the contribution of SRCAP and p400/Tip60 complex subunits to cell division control, we carried out co-immunoprecipitation (co-IP) assays to test interactions at the midbody between two CRS, BAF53a and Tip60, and crucial regulators of cytokinesis. We focused on Alix, anillin, Aurora B, CEP55, citron kinase (CIT-K), MKLP2, and spastin, seven well-known midbody-associated proteins that ensure proper cytokinesis [7, 10–15, 20]. We also tested α-tubulin, a main structural component of spindle and midbody and MRG15.

We choose BAF53a and Tip60 for several reasons: (i) in HeLa and MRC5 cells they both colocalized at the midbody with a sharp fluorescence signal mapping to the central zone (Figs. 2, 3, and 4; Additional file 3: Fig. S3 and Table 1); (ii) their depletion affected cytokinesis, with BAF53a depletion producing a strong effect on the formation of binucleated cells and long intercellular bridges (Fig. 5); (iii) BAF53a has structural features common to actin [23], a contractile ring component required for cytokinesis [36]; (iv) Tip60 is an acetyl-transferase that interacts and acetylates Aurora B in metaphase [33]; (v) both BAF53a and Tip60 coding genes have been implicated in cancer [37, 38]; (vi) The BAF complex subunits are implicated in a group of genetic diseases called BAFopathies [39].

Co-IP assays were performed on chromatin-free protein extracts from the cytoplasmic fraction of HeLa cells synchronized in telophase (see “Methods”), using antibodies against BAF53a and Tip60, previously validated by IFM and WB (Fig. 3). Telophase synchronization was followed by subcellular fractionation assays to recover the cytoplasmic component (S2 fraction) and segregate away the chromatin-associated components (see “Methods”).

**Fig. 6:**
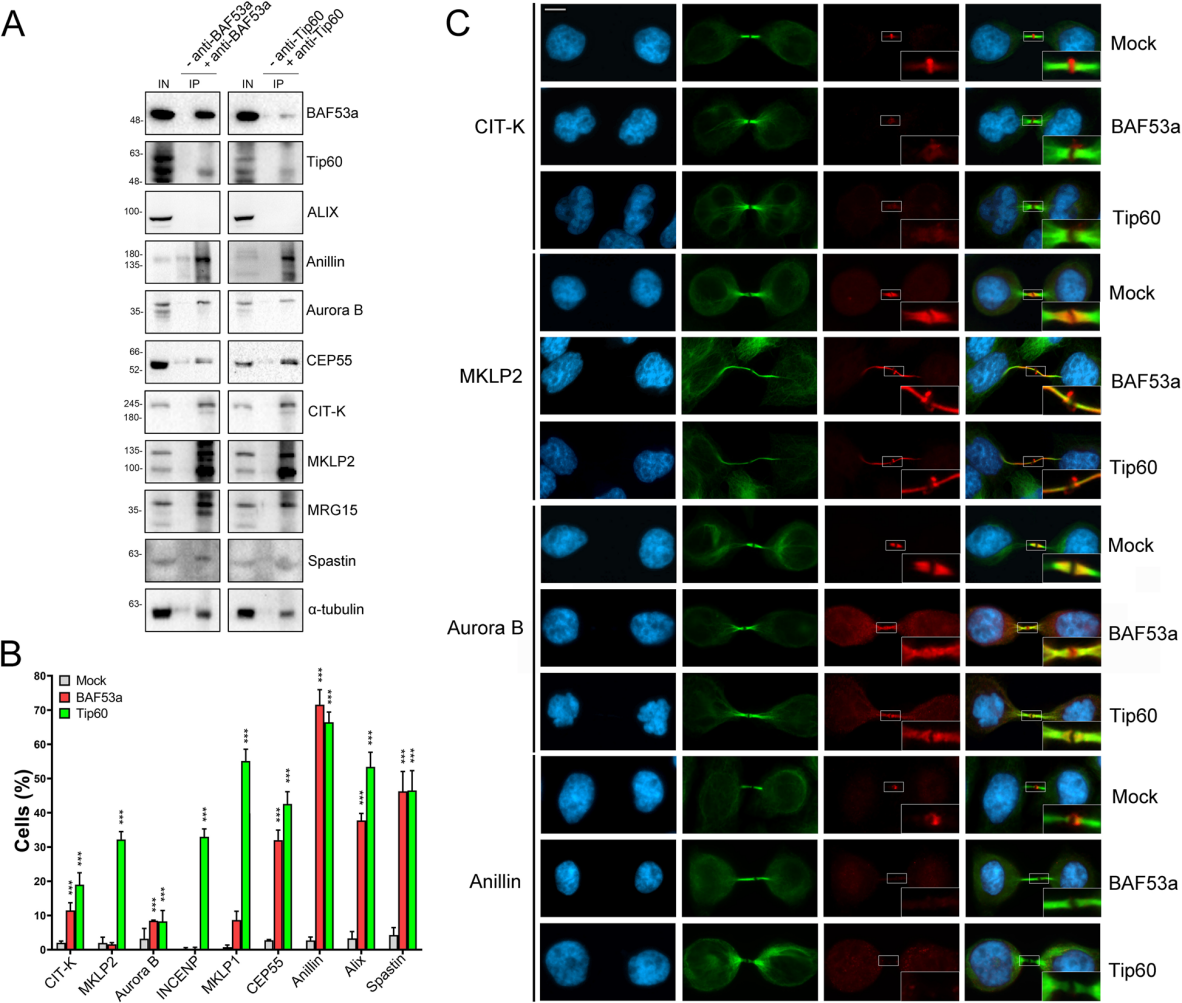
Interactions between CRS and cytokinesis regulators. **A** Immunoprecipitation of protein extracts from cytoplasmic fraction of telophase synchronized HeLa cells (S2 fraction). IP sample immunoprecipitated with BAF53a or Tip60 antibodies (+anti-BAF53a; +anti-Tip60) were compared to negative controls (−anti-BAF53a or −anti-Tip60). Anillin, Aurora B, CEP55, CIT-K, MKLP2, MRG15, spastin, and α-tubulin were found in the IP samples immunoprecipitated with BAF53a or Tip60 antibodies, but not in the negative controls. Three independent IP experiments were performed. IN = input, IP = immunoprecipitation. Synchronization of the HeLa cells was performed according to Messina et al. [31]. **B** Histograms showing the quantitative analysis of mislocalizations of cytokinesis regulators at the midbody in mocks and BAF53a or Tip60 depleted cells. Three independent experiments were performed and at least 300 telophases were scored in both RNAi-treated and control cells. *=P<0.05,**=P<0.005, ***=P<0.0005. **C** Examples of mislocalization of cytokinesis regulators in mock and BAF53a or Tip60 depleted cells. From left to the right: DAPI (blue), anti-α-tubulin (green), cytokinesis regulators (red), and merge. Scale bar = 10 μm

As shown in Fig. 6A, from the comparison between the negative control (−BAF53a and −Tip60) and the IP (+BAF53a and +Tip60), BAF53a and Tip60 interacted with each other, consistent with their co-localization to the midbody (Fig. 2), and with all cytokinesis regulators tested, but Alix. Moreover, both BAF53a and Tip60 interacted with MRG15 and α-tubulin.

### The proper localization of cytokinesis regulators to the midbody is affected by depletion of BAF53a or Tip60

The aforementioned results suggested that BAF53a and Tip60 may contribute to the recruitment of cytokinesis regulators to the midbody, with a role in the regulatory pathway that controls the final step of cytokinesis. To test this hypothesis, we asked whether depletion of BAF53a and Tip60 affects the recruitment at the midbody of the same cytokinesis regulators tested in the co-IP experiments. As shown in Fig. 6B, C and Table 3, the results of three independent replicates showed that the midbody localization pattern of the cytokinesis regulators under investigation was impaired in BAF53a and Tip60 depleted HeLa cells. In particular, a strong alteration of the localization pattern was observed for anillin, Alix, CEP55, MKLP2, and spastin.

**Table 3:** Mislocalization of cytokinesis regulators in CRS-depleted HeLa cells

CR	Mock	BAF53a	Tip60
CIT-K	2.03 ± 0.47	11.44 ± 2.28***	18.99 ± 3.48***
MKLP2	1.94 ± 1.71	1.55 ± 0.49	32.21 ± 2.28***
Aurora B	3.20 ± 3.02	8.47 ± 0.18***	8.25 ± 3.18***
MKLP1	0.73 ± 0.64	8.66 ± 2.57***	55.16 ±3.42***
Cep55	2.77 ± 0.20	32.00 ± 2.94***	42.56 ± 3.59***
Anillin	2.72 ± 0.98	71.58 ± 4.36***	66.44 ± 2.98***
Alix	3.27 ± 2.03	37.75 ± 2.05***	53.39 ± 4.29***
Spastin	4.27 ± 2.18	46.27 ±5.79***	46.51 ± 5.80***

The results are expressed as mean ± SD values from three independent replicate experiments

*CR* cytokinesis regulators

Experimental groups were compared with control groups (mocks) by Fisher's exact test. *=*P* < 0.05; **=*P* < 0.005; and ***P= < 0.0005

### The proper localization of BAF53a and Tip60 to the midbody is affected by the inhibition of Aurora B kinase activity

An important question is how SRCAP and p400/TIP60 chromatin remodelers are recruited to the midbody. The results of our IP assays indicated that BAF53a and Tip60 interact with Aurora B kinase, a key regulator of mitosis and cytokinesis which interacts with several microtubule-binding proteins [10, 14, 34].

**Fig. 7:**
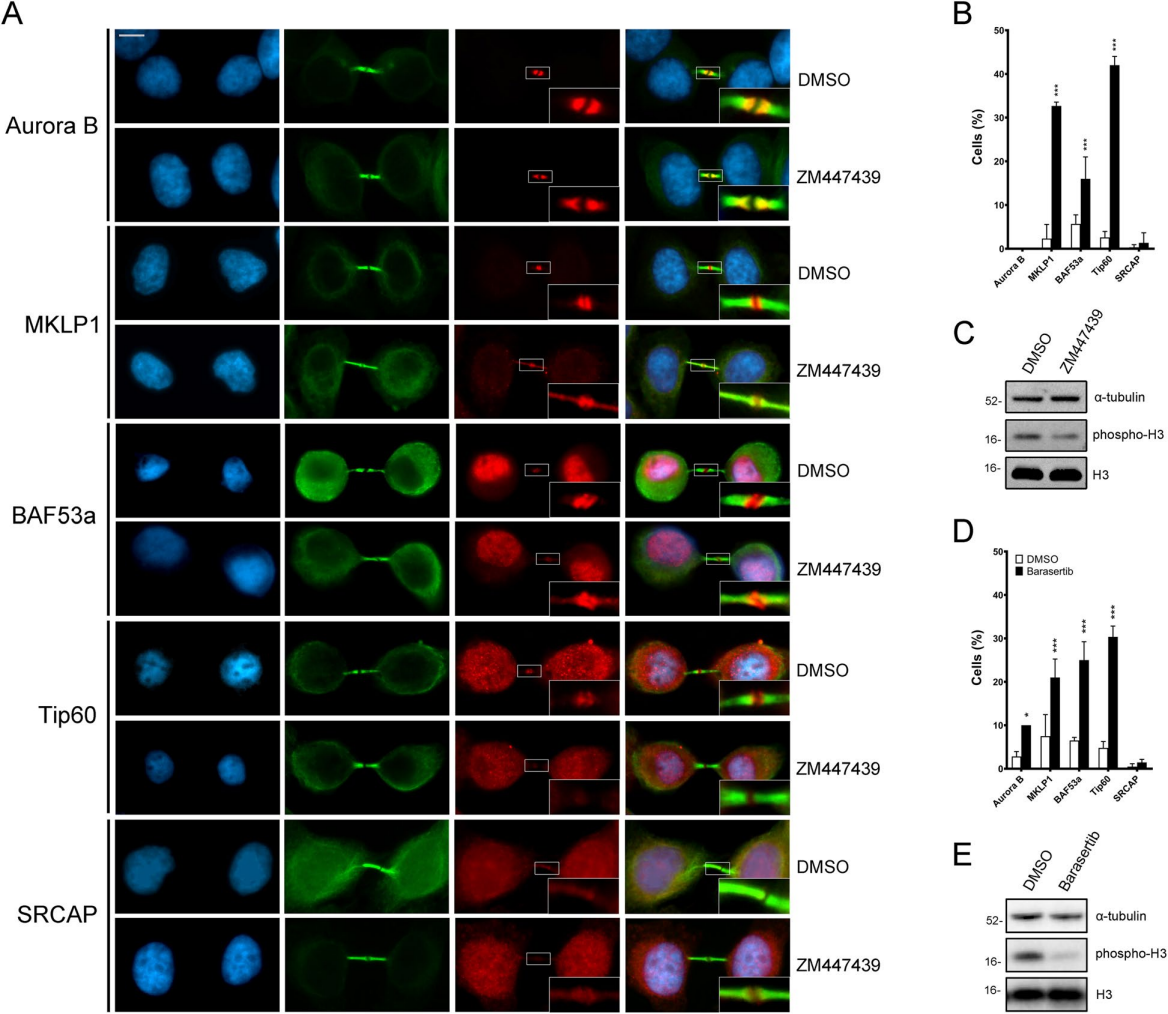
Inhibition of Aurora B kinase activity with ZM447439 and Barasertib affected the localization of BAF53a and Tip60 to the midbody. **A** Treatment with ZM447439: from left to right: DAPI (blue), anti- α-tubulin (green), tested protein (red), and merge. Anti-MKLP1 and anti-Aurora B immunostaining were used as positive and negative controls, respectively. Scale bar = 10 μm. The localization pattern of BAF53a and Tip60, as well as that of MKLP1 were affected, while no effect was seen for SRCAP and Aurora B. **B** The effects found with ZM447439 treatment are summarized in the graph. At least 300 telophases were scored in three independent experiments for both treated cells and control HeLa cells. **C** To test the effectiveness of ZM447439 inhibition, phosphorylation of the histone H3 (target of Aurora B kinase) during mitosis was evaluated; α-tubulin was used as loading control. **D** The effects found with Barasertib treatment are summarized in the graph. At least 200 telophases were scored in two independent experiments for both treated cells and control HeLa cells. A slight effect on Aurora localization was seen at the limit of significance values. **E** To test the effectiveness of Barasertib inhibition, phosphorylation of the histone H3 (target of Aurora B kinase) during mitosis was evaluated; α-tubulin was used as loading control. *=P<0.05, **<=P<0.005, ***=P<0.0005

To understand the relationships between Aurora B and the CRS under investigation, we asked whether the inhibition of its kinase activity affects the recruitment of both BAF53a and TIP60 to the midbody. We also tested the SRCAP ATPase, since it was found to control Aurora B localization to the midbody [31]. To inhibit of the Aurora B kinase activity, the ZM447439, a specific selective ATP-competitive inhibitor was used [40]. We found that the midbody localizations of BAF53a, Tip60, and MKLP1 are significantly affected in HeLa cells treated for 35 min with ZM447439, compared to that in DMSO-treated controls (Fig. 7A–C and Table 4). No effect was seen on Aurora B and on SRCAP localization. The use of Barasertib, another inhibitor of Aurora B kinase activity [41], produced similar results (Fig. 7D, E), the only difference being a slight effect on the localization of Aurora, with a *p*-value at the limit of significance. As control of inhibition efficiency, phosphorylation of the histone H3 during mitosis was assessed (Fig. 7C, E). Together, the results obtained using two different inhibitors strongly suggested that the Aurora B kinase activity is required for proper localization of BAF53a and Tip60 to the midbody and also support the hypothesis of a cross-regulation occurring during cytokinesis between chromatin remodelers and cytokinesis regulators.

**Table 4:** Treatment with ZM447439 (ZM) and Barasertib (BS) inhibitors

	DMSO (ZM)	ZM447439	DMSO (BS)	Barasertib
Aurora B	0.00 ± 0.00	0.00 ± 0.00	2.81 ± 1.14	10.00 ± 0.00*
MKLP1	2.34 ± 3.21	32.68 ± 0.87***	7.50 ± 4.95	21.00 ± 4.24***
BAF53a	5.67 ± 2.08	16.00 ± 5.00***	6.50 ± 0.71	24.50 ± 4.95***
Tip60	2.58 ± 1.37	42.00 ± 3.00***	4.78 ± 1.48	30.34 ± 2.50***
SRCAP	0.34 ± 0.58	1.33 ± 2.31	0.48 ± 0.67	1.43 ± 0.71

The results are expressed as mean ± SD values from three independent replicate experiments

Experimental groups were compared with DMSO-treated controls by Fisher's exact test. *=*P* <0.05; **=*P* < 0.005; and ***=*P* < 0.0005

### The proper localization of BAF53a and CFDP1 to the midbody is affected by SRCAP depletion

**Fig. 8:**
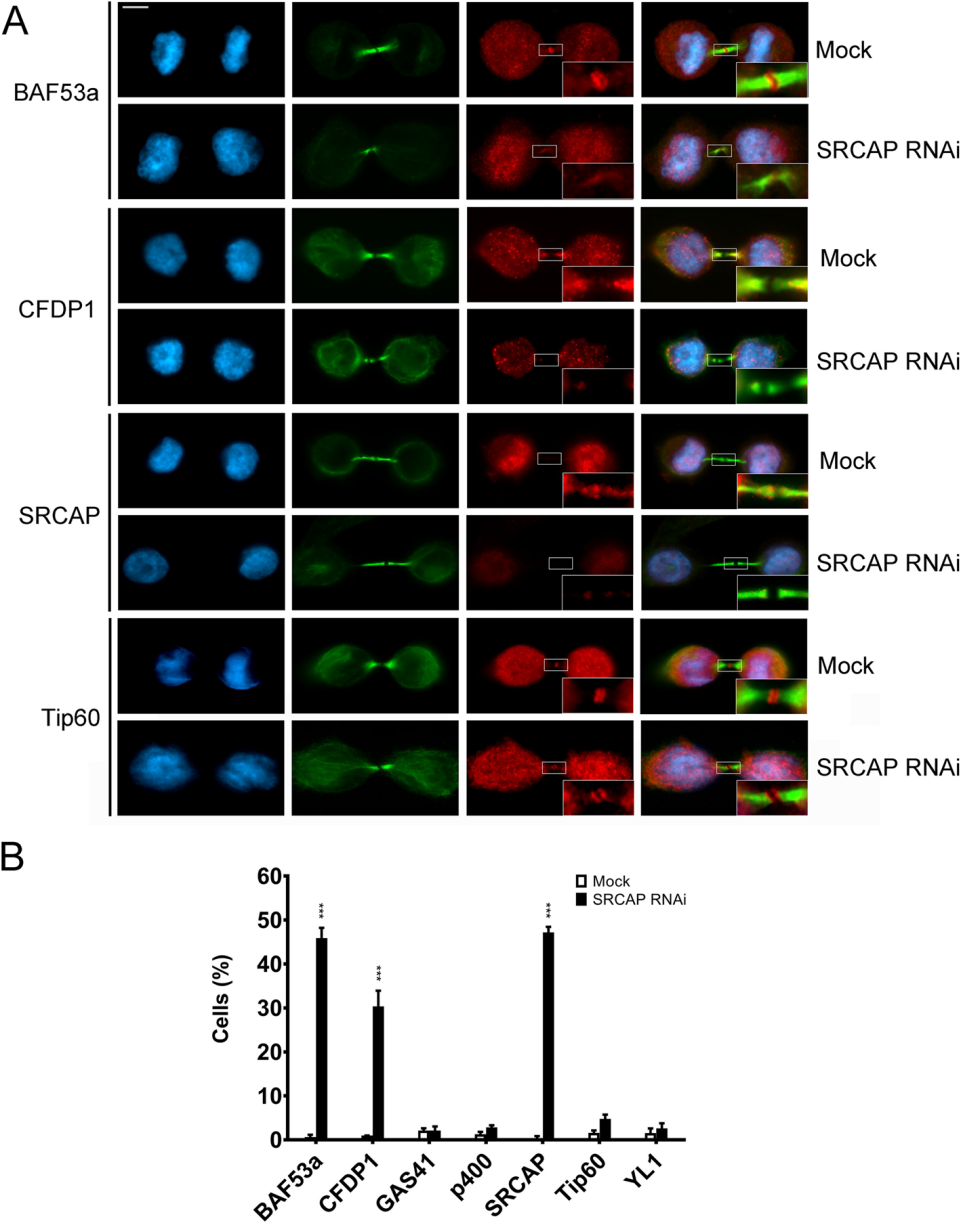
SRCAP-dependent localization of BAF53a and CFDP1 to the midbody. **A** Examples of mislocalization. DAPI (blue), anti-α-tubulin (green), tested CRS (red), and merge. Scale bar = 10 μm. **B** The graph summarized the results. At least of 300 telophases were scored in two independent experiments for both treated cells and control HeLa cells. ***=P<0.0005

A further question regarding the mechanisms underpinning the recruitment of CRS to the mitotic apparatus is whether they maintain their interactions during the relocation or, alternatively, move independently. We have shown that BAF53a and Tip60 colocalize and interact with each other and with MRG15 at the midbody (Figs. 2, 4 and 6), suggesting that the three subunits are still interacting during their “trip” from interphase chromatin to the mitotic apparatus. To further investigate this aspect, we studied the localization to the midbody of CFDP1 (SRCAP complex specific subunit), BAF53a, GAS41, YL1 (subunits shared by both the SRCAP and p400/Tip60 complexes), p400, and Tip60 (p400/Tip60 complex specific subunits) in HeLa cells depleted for SRCAP, the main platform of the homonymous complex. As shown in Fig. 8, SRCAP depletion clearly affects the localization of both CFDP1 and BAF53a, but not that of p400, Tip60, GAS41, and YL1.

### Subcellular localization and RNAi-mediated depletion of MRG15, Tip60, and YETI, three subunits of the *D. melanogaster* Tip60 complex

**Fig. 9:**
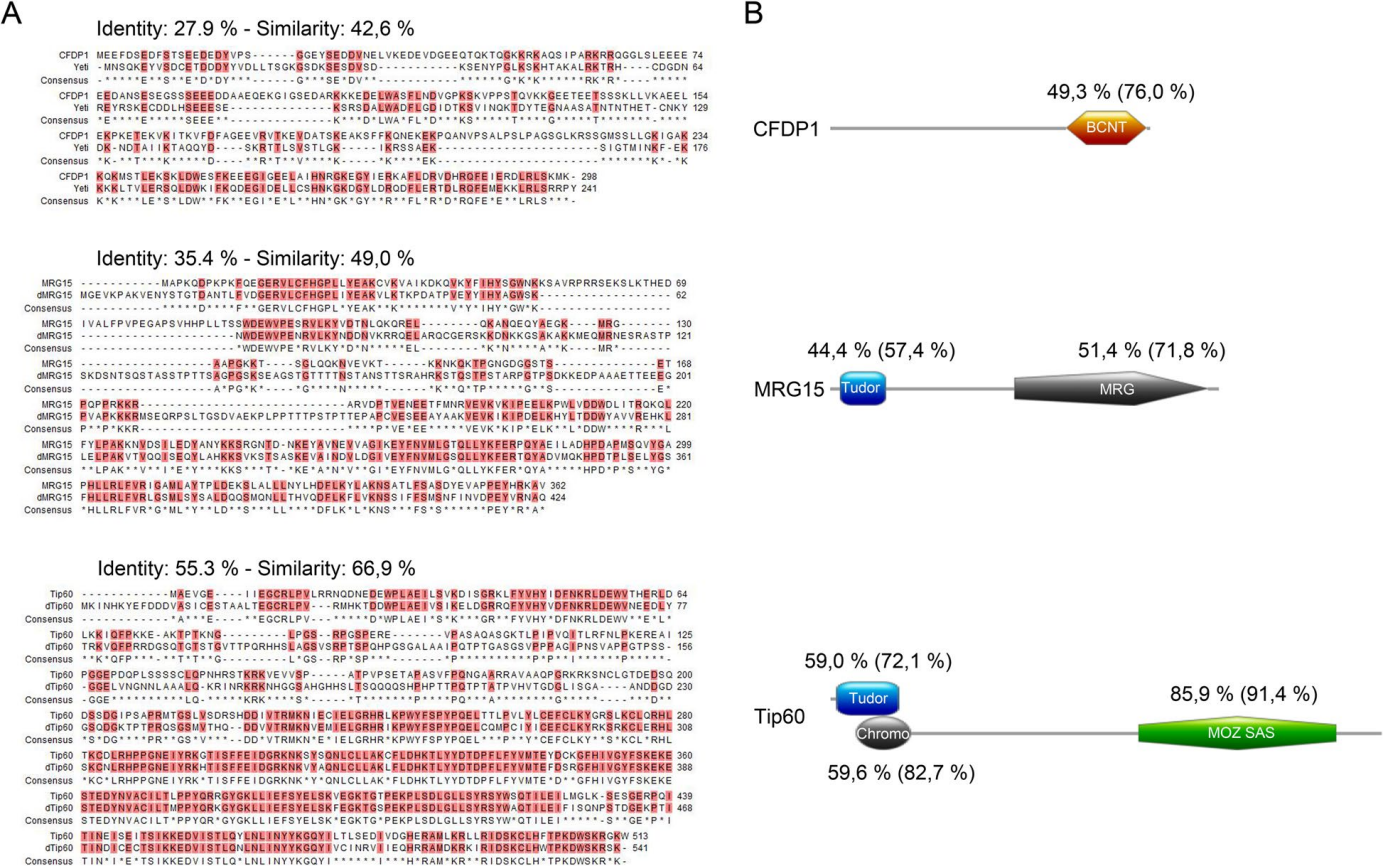
Sequence conservation of *Drosophila* MRG15, dTip60, and YETI proteins with their human orthologs. **A**) BLAST alignments; **B**) High identity levels found in specific functional domains of CFDP1, MRG15 and Tip60

In past studies, we found that the lack of *D. melanogaster* YETI protein affects chromosome organization and impairs cytokinesis [42–46]. YETI is the *Drosophila* ortholog of CFDP1 [47–49] and belongs to the dTip60 chromatin remodeling complex [50] which includes subunits sharing high sequence and function conservation with those of SRCAP and p400/Tip60 complexes. Recently, we found that DOM-A, the functional SRCAP orthologue in *D. melanogaster*, is recruited to centrosomes and midbody in *Drosophila* S2 cells, and its depletion affects both mitosis and cytokinesis [31]. Here, we asked whether this behavior is shared by other dTip60 complex subunits. To answer this question, we used IFM to study the distribution of MRG15, Tip60, and YETI in S2 cells. These subunits are structurally and functionally orthologous to human MRG15, Tip60, and CFDP1, respectively. As shown in Fig. 9, high levels of identity and similarity are found at specific functional domains of these proteins (BCNT, TUDOR, MRG, CHROMO; MOZ_SAS).

**Fig. 10:**
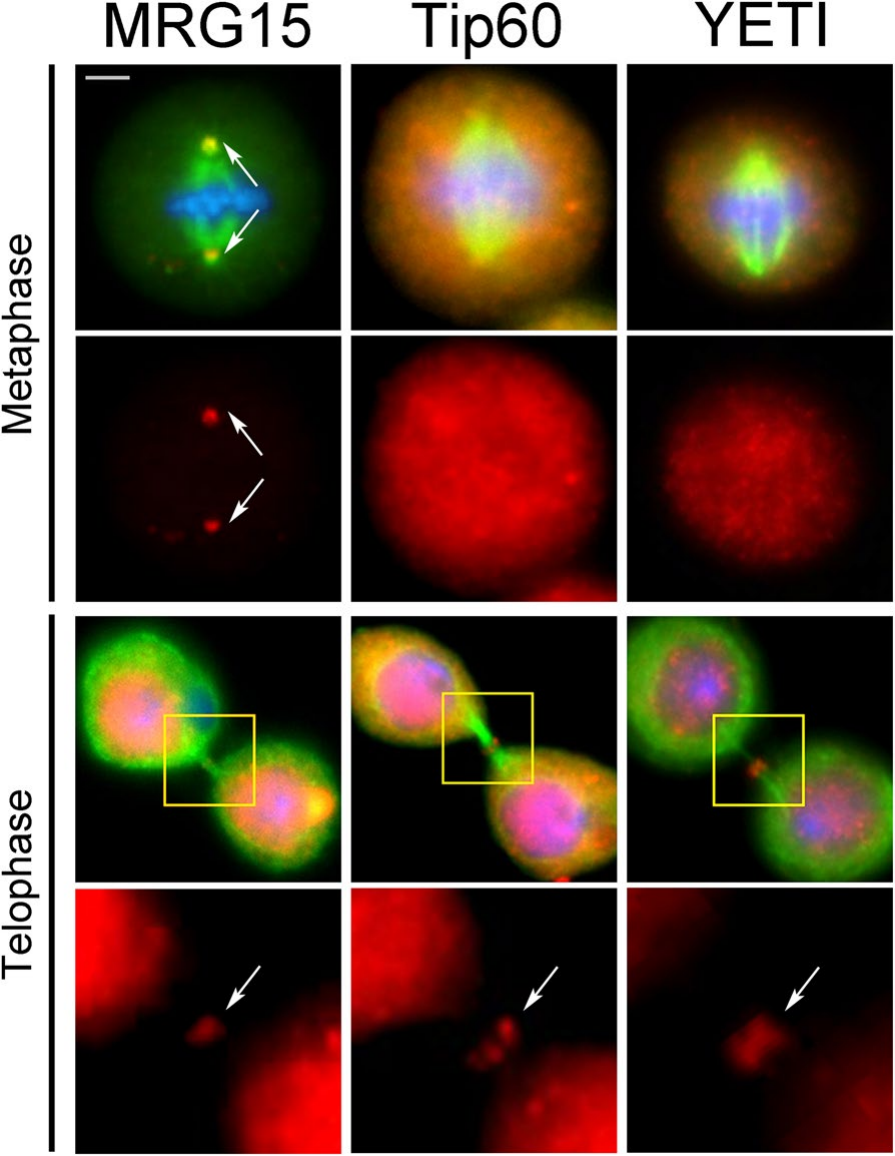
Immunostaining showing the localization of MRG15, Tip60, and YETI to the mitotic apparatus in *D. melanogaster* S2 cells. Cells were stained with DAPI (blue), anti-α-tubulin (green), and anti-MRG15, anti-Tip60, and anti-YETI (red). Scale bar = 5 μm. In addition to interphase nuclei, the anti-MRG15 decorated the centrosomes (metaphase) and midbody (telophase), while anti-Tip60 and anti-YETI staining was detected on the midbody

The results of this experiment showed that MRG15, Tip60, and YETI, apart from to their canonical location in chromatin, are found at the midbody (Fig. 10), similarly to their human orthologs. In addition, MRG15 was localized to the centrosomes. Thus, the recruitment of CRS to the mitotic apparatus appears to be an evolutionary conserved phenomenon.

**Fig. 11:**
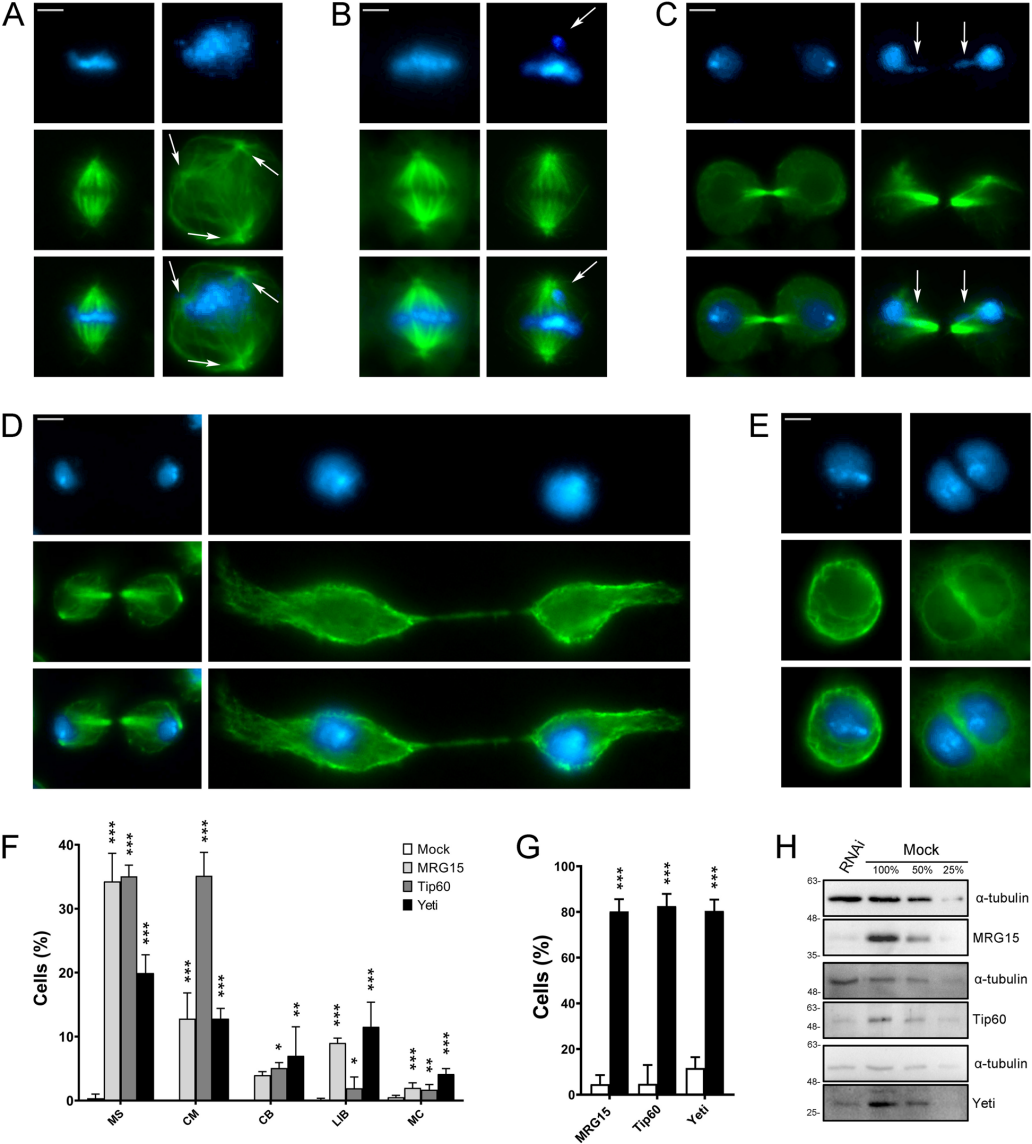
RNAi-mediated depletion of MRG15, Tip60, and YETI in S2 cells affects mitosis and cytokinesis. Examples of mitotic defects found in MRG15, Tip60, and YETI depleted S2 cells and their quantification. DAPI staining is shown in blue, α-tubulin in green. RNAi knockdown experiments were performed by transfecting S2 cells with specific siRNAs against each protein (see “Methods”). Scale bar = 5 μm. Five classes of defects were considered: **A** multipolar spindle (MS); **B** chromosome misalignments in metaphase (CM); **C** chromatin bridges (CB); **D** long intercellular bridges (LIB); **E** multinucleated cells (MC); **F** Quantitative analysis of defects scored in RNAi-treated and control cells (Table 5) is based on the following numbers: at least 100 prometaphases and metaphases for MS, 70 metaphases for CM, 300 telophases for LIB and CB, 5500 interphases for MC. Three independent experiments were performed. **G** Percent of cells showing a decrease of fluorescence intensity at the midbody in MRG15, Tip60, or YETI depleted cells (black histograms) compared to the mocks (white histograms). The results are based on three independent experiments. **H** WB showing a decrease of total amount of MRG15, Tip60, and YETI proteins in RNAi-treated S2 cells compared to the mocks; a-tubulin is used as loading control

Next, we examined the phenotypes of S2 cells after RNAi against MRG15, Tip60, and YETI. The depletion of these subunits indeed resulted in five categories of defects (Fig. 11A–F and Table 5) comparable to those observed in HeLa cells depleted for their corresponding orthologs: multipolar spindles (MS), chromosome misalignments in metaphase (CM), chromatin bridges (CB), long intercellular bridges (LIB), and multinucleated cells (MC). The actual effectiveness of RNAi treatments was tested by IF and WB assays (Fig. 11G, H). The depletion of MRG15, Tip60, and YETI affected mitosis in terms of cells with MS and CM. Mild and yet statistically significant effects were seen on CB. MRG15 and YETI depletion also affected abscission with 9% and 11.54% of LIB, respectively. Finally, YETI depletion resulted in 4% of MC.

**Table 5:** Quantitative analysis of defects found in CRS-depleted S2 cells

		Mock	MRG15	Tip60	Yeti
Metaphase	MS	0.37 ± 0.64	34.28 ± 4.39***	35.06 ± 1.75***	19.94 ± 2.84***
	CM	0	12.79 ± 4.03***	35.16 ± 3.66***	12.81 ± 1.60***
Telophase	CB	0	3.97 ± 0.55	5.08 ± 0.85*	6.99 ± 4.54**
	LIB	0.13 ± 0.22	9.02 ± 0.74***	1.92 ± 1.76*	11.54 ± 3.84***
	MC	0.53 ± 0.29	1.98 ± 0.79***	1.70 ± 0.80**	4.14 ± 0.84***

*MS* multipolar spindles, *CM* chromosome misalignments, *CB* chromatin bridges, *LIB* long intercellular bridges, *MC* multinucleated cells

The results are expressed as mean ± SD values from three independent replicate experiments

Experimental groups were compared with mocks by Fisher's exact test. *=*P* < 0.05; **=*P* < 0.005; and ***=*P* < 0.0005

## Discussion

### Evolutionary conserved relocation of CRS to the mitotic apparatus

The first example of proteins that change their localizations and functions during cell cycle progression was given by Aurora B, INCENP, Borealin, and Survivin, members of the so-called chromosome passenger complex (CPC). In early mitosis, they associate with chromosomes and then relocate to the spindle and midbody, playing essential roles in cell division [51]. Moreover, INO80, Pontin, Reptin, and Tip60 chromatin remodelers were shown to relocate to the mitotic apparatus and suggested to play direct roles in cell division in human cells [24–29]. Recently, we have found that the SRCAP ATPase, apart from its canonical localization and roles on chromatin, relocates to the centrosomes, spindle and midbody, interacts with α-tubulin and with several cytokinesis regulators, positively regulating their recruitment to the midbody [31]. Moreover, the epigenetic factor PBRM1, a subunit of the PBAF chromatin remodeling complex, was found to be recruited to microtubules and required for proper mitosis [52].

Here, we combined cell biology, functional genomics and biochemical approaches to deepen our understanding of the roles played by subunits of human SRCAP and p400/TIP60 chromatin remodeling complexes during mitosis and cytokinesis. We have shown that during cell cycle progression seven subunits of SRCAP and p400/Tip60 complexes relocate to centrosomes, spindle, and midbody in human cell lines (Figs. 2 and 3 and Additional file 3: Fig. S3). Working on isolated midbodies (Fig. 4A, B), we confirmed the midbody associations of CRS found on fixed cells and also those of Pontin and Reptin [24–26, 29]. In addition, we found an extrachromosomal localization of histone H2A.Z to the isolated midbodies. H2A.Z is the target of SRCAP complex activity [23], and its localization to the midbody is intriguing, because it resembles that of histone H2B which is recruited to the midbody by Aurora B and is required for proper cytokinesis [53, 54].

RNAi knockdown experiments showed that the depletion of each subunit tested yields an array of aberrant mitotic outcomes in HeLa cells (Fig. 5 and Table 2). Most notably, BAF53a and Tip60 interacted at telophase with each other and with a number of cytokinesis regulators, positively controlling their recruitment to the midbody (Fig. 6 and Table 3).

In parallel, we found that MRG15, Tip60, and YETI are recruited to the midbody in *D. melanogaster* S2 cells, with MRG15 also localized to the centrosomes (Fig. 10) and their depletion affecting both mitosis and cytokinesis (Fig. 11 and Table 5).

Notably, depletion of different subunits belonging to the same complex did not produce the same results (Fig. 5 and Table 2). It is possible that the lack of certain subunits has a minor impact on cell division compared to others. Alternatively, a more trivial explanation would assume a different efficiency of the siRNAs targeting to different subunits.

### Functional relevance of CRS in ensuring proper cell division

The observed relocation of CRS to the mitotic apparatus during cell cycle progression may be interpreted as result of passive accumulation of proteins to be disposed of. Under this hypothesis, the cell division defects observed following their depletion could be merely secondary effects caused by general chromatin perturbations, that in turn would result in deregulation of cell division genes. Alternatively, the CRS studied here could be functional components of the mitotic apparatus playing chromatin-independent roles on mitosis and cytokinesis. That the massive association of CRS with mitotic apparatus may have functional relevance is supported by the following considerations.

Firstly, the recruitment of CRS to mitotic apparatus and the following disruption of cell division after their depletion, appears to be a very ancient and evolutionary conserved phenomenon, since it was observed in both humans and *Drosophila melanogaster*, whose lineages separated approximately 780 million years ago [55].

Secondly, the defects observed after depletion of CRS in HeLa and *Drosophila* S2 cells do not seem to be simply a chaotic disruption of cell division caused by simultaneously misexpression of cell division genes. In contrast, specific classes of mitosis and cytokinesis alterations were observed, including multipolar spindle, chromosome misalignments at metaphase, multinucleated cells, and long intercellular bridges (Figs. 5 and 11). Those defects are consistent with the localization of the CRS of interest to the mitotic apparatus and also occur after the loss of known spindle and/or midbody regulators [4–7, 10–15, 53].

Finally and most importantly, a direct role of CRS in cytokinesis is supported by the results of our IFM and co-IP experiments performed on chromatin-free protein extracts from telophase-synchronized HeLa cells: (i) BAF53a and Tip60 were found to interact at telophase with α-tubulin and with Aurora B, CIT-K, and other midbody-associated proteins that play crucial roles in cytokinesis regulation (Fig. 6A), with the midbody recruitment of the same regulators depending on BAF53a and Tip60 activity (Fig. 6B,C); (ii) the Aurora B kinase activity promoted the recruitment of BAF53a and Tip60 to the midbody (Fig. 7). Notably, an interaction between BAF53a and CIT-K has also been highlighted in a recent study on the midbody interactome [8].

All the proteins identified here as BAF53a and Tip60 interactors in telophase are evolutionary conserved cytokinesis regulators whose depletion results in aberrant cytokinesis phenotypes in distantly related species. CIT-K is the main abscission regulator physically and functionally interacting with the actin-binding protein anillin, a crucial component of the contractile ring and of the midbody [8]; CIT-K is also required for localization of F-actin and anillin at the abscission sites [7, 56, 57]. MKLP2 is a motor kinesin that binds microtubules and is required for Aurora B recruitment at the central spindle [10]. CEP55 recruits Alix at the midbody and in its absence, a series of late-acting abscission factors fail to concentrate at the midbody, including Aurora B, MKLP2, Plk1, PRC1, ECT2, and the ESCRT machinery [58]. Spastin is a key player in microtubule severing, ensuring the final cut at the midbody [11, 59], whereas α-tubulin is a major component of spindle and midbody microtubules. Notably, mutations in genes encoding tubulins or functionally related proteins such as CIT-K and spastin trigger the onset of neurodevelopmental disorders, including microcephaly [60–63]. In this context, we speculate that during the final stage of cell division the actin-like BAF53a, as midbody component, participates in the recruitment/stabilization of cytokinesis regulators at the midbody, ensuring the final cut essential for abscission. Thus, cytokinesis failure may be an important causative factor co-occurring at the onset of BAFopathies [39] and other neurodevelopmental disorders.

We also showed that BAF53a and Tip60 interacted with each other and with MRG15 at the midbody (Fig. 6). Moreover, SRCAP positively controlled the midbody recruitment of both CFDP1 and BAF53a (Fig. 8). It must be remembered that BAF53a belongs to both SRCAP and p400/Tip60 complexes. These findings suggest that some CRS retain their physical interactions across cell cycle progression and relocate as sub-complexes. However, the mechanisms underpinning removal of the CRS from chromatin and transport to the mitotic apparatus remain unclear.

Interactions of CRS such as INO80, Pontin, Gas41, SRCAP, and others with tubulins were detected in previous studies [24, 26, 27, 31, 52, 64, 65]. Here, we found that BAF53a, Tip60, and CFDP1 interact with α-tubulin (Fig. 6 and Supplementary Fig. 4). It is then possible to speculate that the CRS undergo relocation as subcomplexes by exploiting the interactions with microtubules and/or microtubule-associated proteins. Exploring this aspect will be an important topic for future analyses.

## Conclusions

Overall, our results disclosed the existence of a massive and evolutionarily conserved phenomenon, whereby subunits of SRCAP and p400/Tip60 complexes relocate to the mitotic apparatus and have functional relevance in cell division. These proteins function beyond chromatin remodeling and are leading a “double life”: on the one hand, as chromatin factors they play canonical roles in the epigenetic regulation of gene expression, and on the other, as component of the mitotic apparatus ensure the fidelity of mitosis and cytokinesis, preventing genetic instability states. Indeed, mutations in genes encoding the subunits of SRCAP and p400/Tip60 complexes result in tumorigenesis [37, 38, 66–72]. Thus, our results also highlight an interesting scenario in which chromatin remodeling, cell cycle, tumorigenesis, and developmental diseases are closely interlinked.

A “moonlighting protein” is a protein found in unexpected locations within the cell, performing one or more additional functions besides the canonical one [73, 74]. Yokoyama highlighted the moonlighting function of a number of chromatin-binding proteins as mitotic microtubules regulators [65]. Here, we propose that the subunits of SRCAP and p400/Tip60 chromatin remodeling complexes are a novel class of moonlighting proteins playing evolutionary conserved roles in mitosis and cytokinesis.

## Methods

### Cell cultures, transfections, and RNAi treatments

HeLa cells (ATTC company) were cultured in 6-well plates in Dulbecco’s modified Eagle’s medium (DMEM) supplemented with 10% FBS (Corning) and a penicillin/streptomycin solution (Gibco, 15140122). RNAi-mediated depletion of CRS was performed using transfection with specific siRNA mixes ([31]; Additional file 6: Table S1). As negative controls, cells were transfected with scrambled siRNA (CAUCGAGACGCUAGCAGAUCCUGCG), already validated [31] or processed excluding the addition of siRNA (mock). The Lipofectamine RNAi-MAX reagent (Thermo Scientific) was used for transfections, according to the manufacturer’s protocol; 24 h after the second transfection, cells were harvested for cytological and immunoblotting analysis. *Drosophila melanogaster* S2 cells were cultured at 25 °C in Schneider’s *Drosophila* Medium (Biowest). RNAi treatments were carried out according to our previous work [31]. To perform *Drosophila* MRG15, Tip60, and YETI protein depletion, each culture was inoculated with 15 μg of specific siRNA targeting the *Mrg15*, *Tip60*, and *Yeti* genes, respectively. Control samples were treated in the same way without addition of dsRNA. Both dsRNA-treated and control cells were grown for 96 h at 25 °C and then processed for either immunofluorescence or blotting analysis. To prepare dsRNA, individual gene sequences were amplified by PCR from genomic DNA obtained from first-instar larvae of a wild type *D. melanogaster* strain. The primers used in the PCR reactions were 48 nt base long and all contained a 5′ T7 RNA polymerase binding site (5′-GAATTAATACGACTCACTATAGGGAGAC-3′) joined to MRG15-, dTip60-, and Yeti-specific sequences, respectively. The sense and antisense gene-specific primers are listed in Additional file 7: Table S2.

### Immunofluorescence

For immunofluorescence (IF) staining, HeLa cells were seeded on glass coverslips, and 24 h later, they were fixed for 15 min at room temperature (RT) in 2% formaldehyde in PBS. After permeabilization in 0.2% Triton X-100 solution and washing in PBS, the cells were incubated in 3% bovine serum albumin (BSA) for 1 h and, subsequently, with primary antibodies overnight at 4 °C. After several washes in PBS, the cells were incubated with secondary antibodies for 1 h at RT. After washing in PBS, the coverslips were counterstained with DAPI and mounted onto slides with ProLong™ Diamond Antifade Mountant (Invitrogen). Preparations were analyzed using a computer-controlled Nikon Eclipse 50i epifluorescence microscope equipped with a CCD camera.

For quantitative analysis of immunofluorescence images, a dedicated tool of Fiji software was used. In brief, a mask was automatically created on α-tubulin signal and manually adjusted by setting up a proper threshold to eliminate the noise. The mask was converted in a selection to apply on remodeler signal which needed to quantify. A set of 5 random background areas from the same image were used to normalize the integrated intensity of the specific signal.

### Antibodies

Primary antibodies and fluorophore- or HRP-conjugated secondary antibodies used for IFM, WB, and IP experiments are described in Additional file 8: Table S3 and Additional file 9: Table S4, respectively.

### Cell cycle synchronization and subcellular fractionation assay

For immunoprecipitation experiments, HeLa cells were synchronized in telophase using thymidine/nocodazole blocks. Cells were treated with 2 mM thymidine (Sigma, T9250) for 19 h, released from G1/S block in fresh media for 5 h, incubated with 40 nM nocodazole (Sigma, M1403) for 13 h, and harvested by mitotic shake-off. Mitotic cells were washed three times with PBS and released in fresh medium for 70 min before harvesting and freezing in liquid nitrogen [31]. Telophase cells (2 × 10^7^) were prepared by resuspending in 1 mL of Buffer A for subcellular fractionation according to Messina et al. [48].

### Inhibition of Aurora B kinase activity

Asynchronous HeLa cells were treated for 35 min with the Aurora B inhibitor ZM447439 (SignalChem, A31-901-01) and Barasertib (AZD1152-HQPA, Selleckchem, S1147) at a final concentration of 5 μM and 200 nM, respectively, and then fixed and stained. An equal volume of DMSO was used as control. The anti-Ser10 H3P was used to test the decreased levels of phosphorylated H3 due to the inhibition of Aurora B kinase activity).

### Western blotting and immunoprecipitations

Western blotting was performed and immunoprecipitation according to our previous work [31]. For immunoprecipitation, rabbit polyclonal antibodies (Abcam) against BAF53a or Tip60 were used. The cytosolic fraction (2 mg/ml) from subcellular fractionation assay was used as input (IN). As negative control, no antibody was added to a same amount of IN and beads (Santa Cruz Biotechnology).

### Midbody isolation

Midbody isolation was performed according to our previous work [31]. IFM and Western blotting were performed by using specific antibodies against the CRS under investigation (Additional file 8: Table S3 and Additional file 9: Table S4), as described in the above paragraphs.

### Microscopy

Both human and *Drosophila melanogaster* slides were analyzed using a computer-controlled Nikon Eclipse 50i epifluorescence microscope equipped with UV-1A EX 365/10 DM 400 BA 400, FITC EX 465-495 DM 505 BA 515-555, and TRITC EX 540/25 DM 565 BA 605/55 filters using Plan Achromat Microscope Objective 100XA/1.25 Oil OFN22 WD 0.2 objective and QImaging QICAM Fast 1394 Digital Camera, 12-bit, Mono. Images were imported into ImageJ software (http://rsbweb.nih.gov/ij/) and adjusted for brightness and contrast uniformly across entire fields where appropriate. The figures were constructed in Adobe Photoshop.

### Bioinformatic analysis

Pairwise sequence alignment was performed using EMBOSS Needle [75] and QIAGEN CLC Main Workbench 21.0.1 (https://digitalinsights.qiagen.com/). Identification, annotation, and graphic output of protein domain were performed using SMART [76] and PROSITE (https://www.expasy.org/resources/prosite).

### Statistical analysis

Data analyses were performed using the GraphPad Prism softwares (GraphPad Software, Inc., La Jolla, CA, USA). All results are expressed as mean±SD values from three independent replicate experiments. *P* value of less than 0.05 (*P < 0.05, **P < 0.005; and ***P < 0.0005 compared with the control group) is considered to be statistically significant by using two-tailed Fisher’s test, and *T*-tests were required [77–79].

## Supplementary Information

**Additional file 1: Figure S1**. Localization of CRS on interphase nuclei. Fixed HeLa cells stained with DAPI (blue), antibody against a give subunit (red) and anti-α-Tubulin (green). As expected, the antibody staining decorated the interphase nuclei. Scale bar = 10 μm. All authors read and approved the final manuscript”.

**Additional file 2: Figure S2**. Validation of antibodies against the CRS. The fluorescence intensity of CFDP1, GAS41, P400 and YL1 decreased in RNAi-treated compared to that of mock-treated cells. The arrows mark the midbody region. Scale bar = 10 μm. Fluorescence intensity was assessed using the ImageJ software and statistical significance was verified by T- test.

**Additional file 3: Figure S3**. Localization of CRS to mitotic apparatus in MRC5 cells. Fixed MRC5 cells stained with DAPI (blue), antibody against the CRS of interest (red) and anti-α-tubulin (green). CFDP1 and YL1 localized to the spindle, while Tip60 the centrosomes in metaphase and anaphase; BAF53a and GAS41 localized to the central spindle in anaphase. All the subunits, with the exception of GAS41, were found at the midbody. Scale bar = 10 μm.

**Additional file 4: Figure S4**. Depletion of CFDP1 in HeLa cells and interactions with a-tubulin. A) Fixed HeLa cells stained with DAPI (blue), anti-a-tubulin (red) CREST antibody (green). Scale bar = 10 μm. B) Histograms showing the % of HeLa cells with monopolar spindle after CFDP1 depletion (black histogram), compared to the control (white histogram); Three independent experiments were performed. The quantitative analysis of defects scored in RNAi-treated and control cells is based on the following numbers: at least 100 prometaphases and metaphases. Experimental group were compared with mock by Fisher's exact test. ***=*P* < 0.0005 C) Immunoprecipitation of EGFP::α-tubulin from asynchronized whole cell extracts. IP sample from stable EGFP::α-tubulin/mCherry::H2B HeLa cells immunoprecipitated with GFP-Trap were compared to negative controls (sample from HeLa cells). CFDP1 and Pontin (positive control) were found in the IP from EGFP::α-Tubulin/mCherry::H2B, but not in HeLa cells samples. Three independent IP experiments were performed. IN = input, FT= Flow Through, IP = immunoprecipitation.

**Additional file 5: Figure S5**. Intercellular distance. Depletion of CRS increased the intercellular distance during cytokinesis. Colors are referred to Fig. 1B. Three independent experiments were performed; statistical significance was verified by T- test.

**Additional file 6: Table S1**. List of siRNAs (Human).

**Additional file 7: Table S2**. Oligos for siRNAs synthesis (Drosophila S2 cells).

**Additional file 8: Table S3**. List of primary antibodies.

**Additional file 9: Table S4**. List of secondary antibodies.

**Additional file 10: Table S4**. Raw data for Tables 2, 3, 4 and 5.

**Additional file 11.** Uncropped blots Fig. 3A.

**Additional file 12.** Uncropped gels/blots Fig. 4B.

**Additional file 13.** Uncropped gels/blots Fig. 6A.

**Additional file 14.** Uncropped gels/blots Fig. 7C, E.

**Additional file 15.** Uncropped gels/blots Fig. 11H.

## Data Availability

All data generated or analyzed during this study are included in this published article and its supplementary information files.
