# Supplementary figures and images for: Evolutionary conserved relocation of chromatin remodeling complexes to the mitotic apparatus
Source: BMC Biol. 2022 Aug 3;20:172. doi: 10.1186/s12915-022-01365-5 (PMC9351137; doi:10.1186/s12915-022-01365-5)

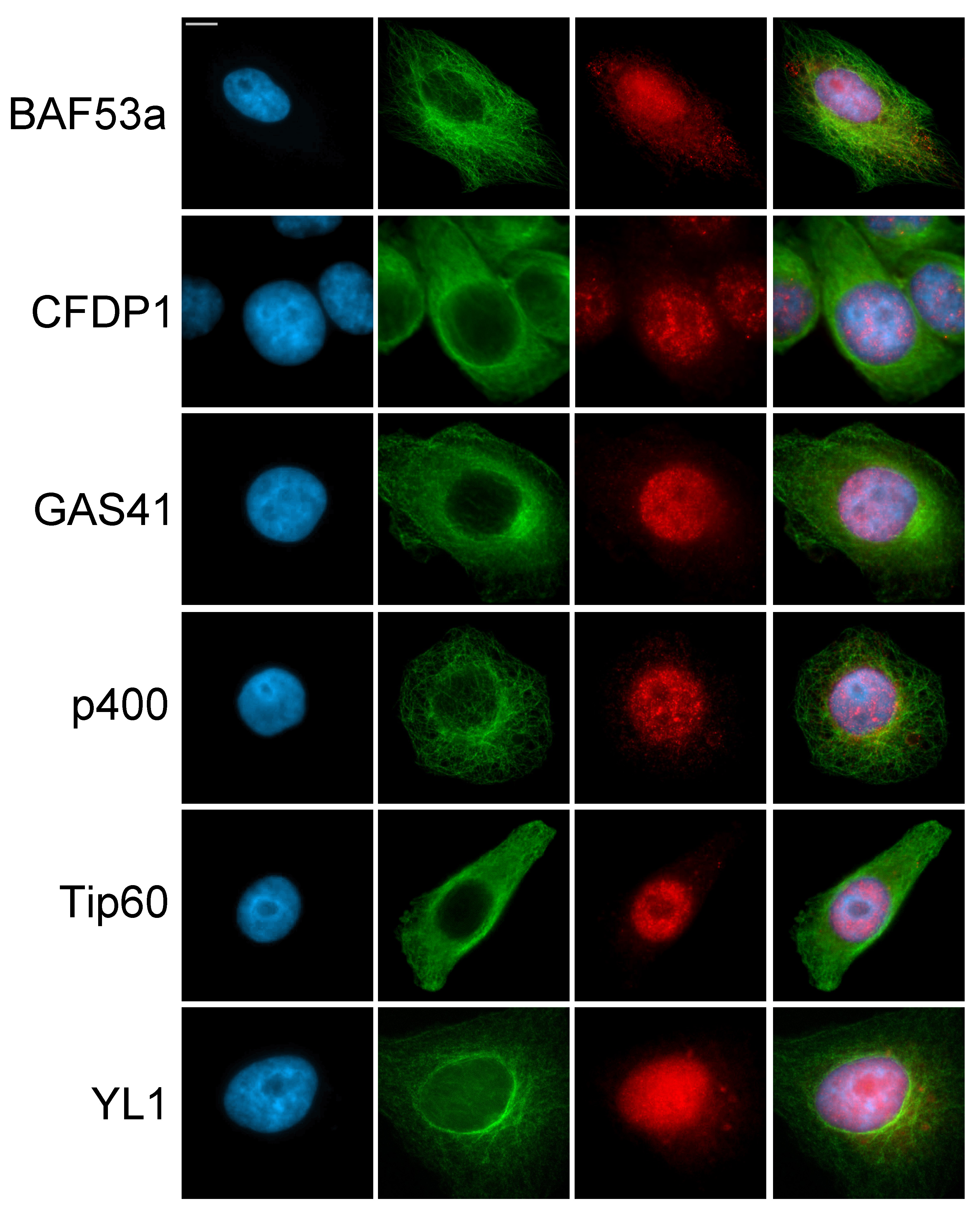

Supplement: Supplementary file 1 — Additional file 1: Figure S1. Localization of CRS on interphase nuclei. Fixed HeLa cells stained with DAPI (blue), antibody against a give subunit (red) and anti-α-Tubulin (green). As expected, the antibody staining decorated the interphase nuclei. Scale bar = 10 μm. All authors read and approved the final manuscript”. [file 12915_2022_1365_MOESM1_ESM.jpg]

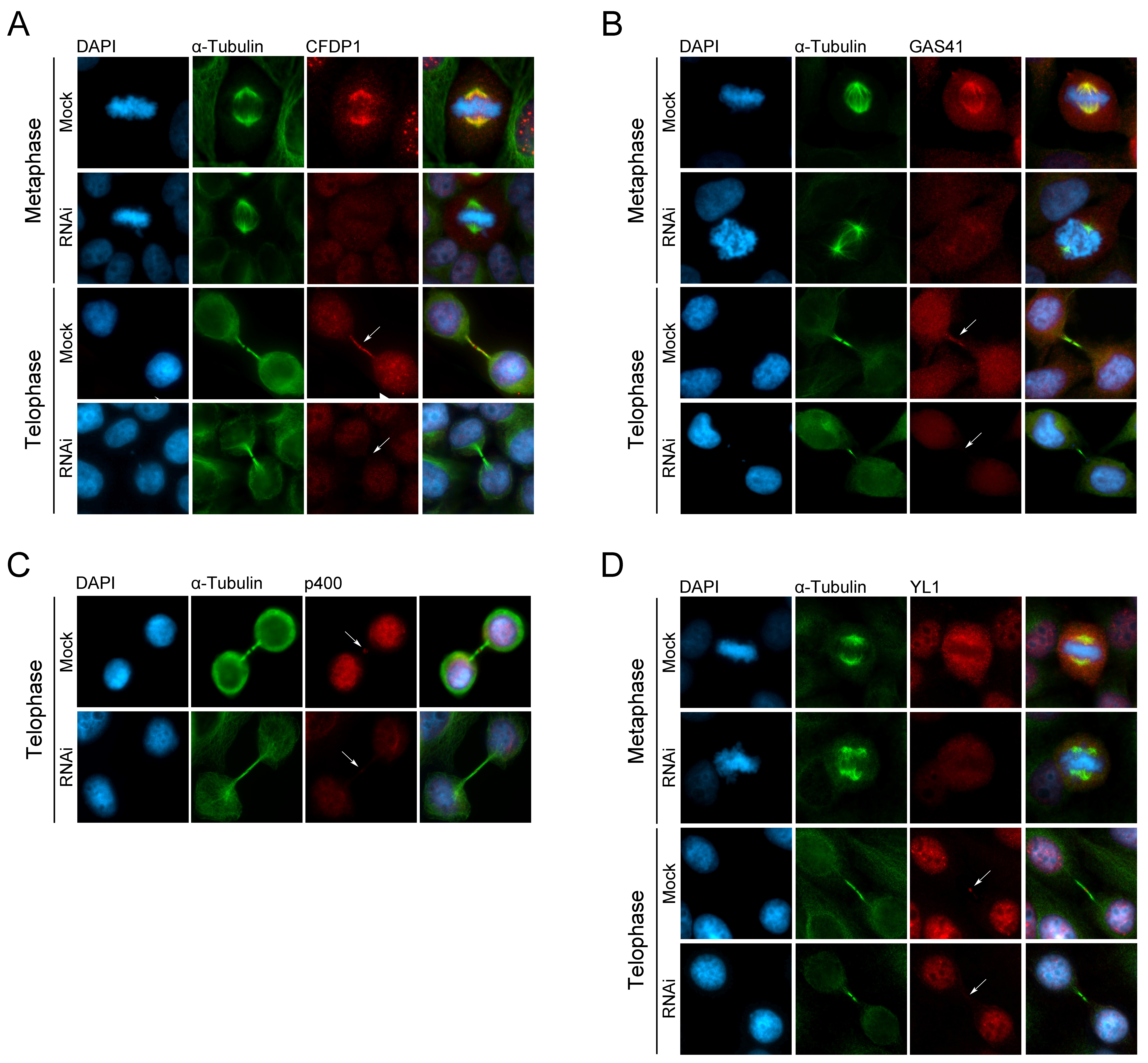

Supplement: Supplementary file 2 — Additional file 2: Figure S2. Validation of antibodies against the CRS. The fluorescence intensity of CFDP1, GAS41, P400 and YL1 decreased in RNAi-treated compared to that of mock-treated cells. The arrows mark the midbody region. Scale bar = 10 μm. Fluorescence intensity was assessed using the ImageJ software and statistical significance was verified by T- test. [file 12915_2022_1365_MOESM2_ESM.jpg]

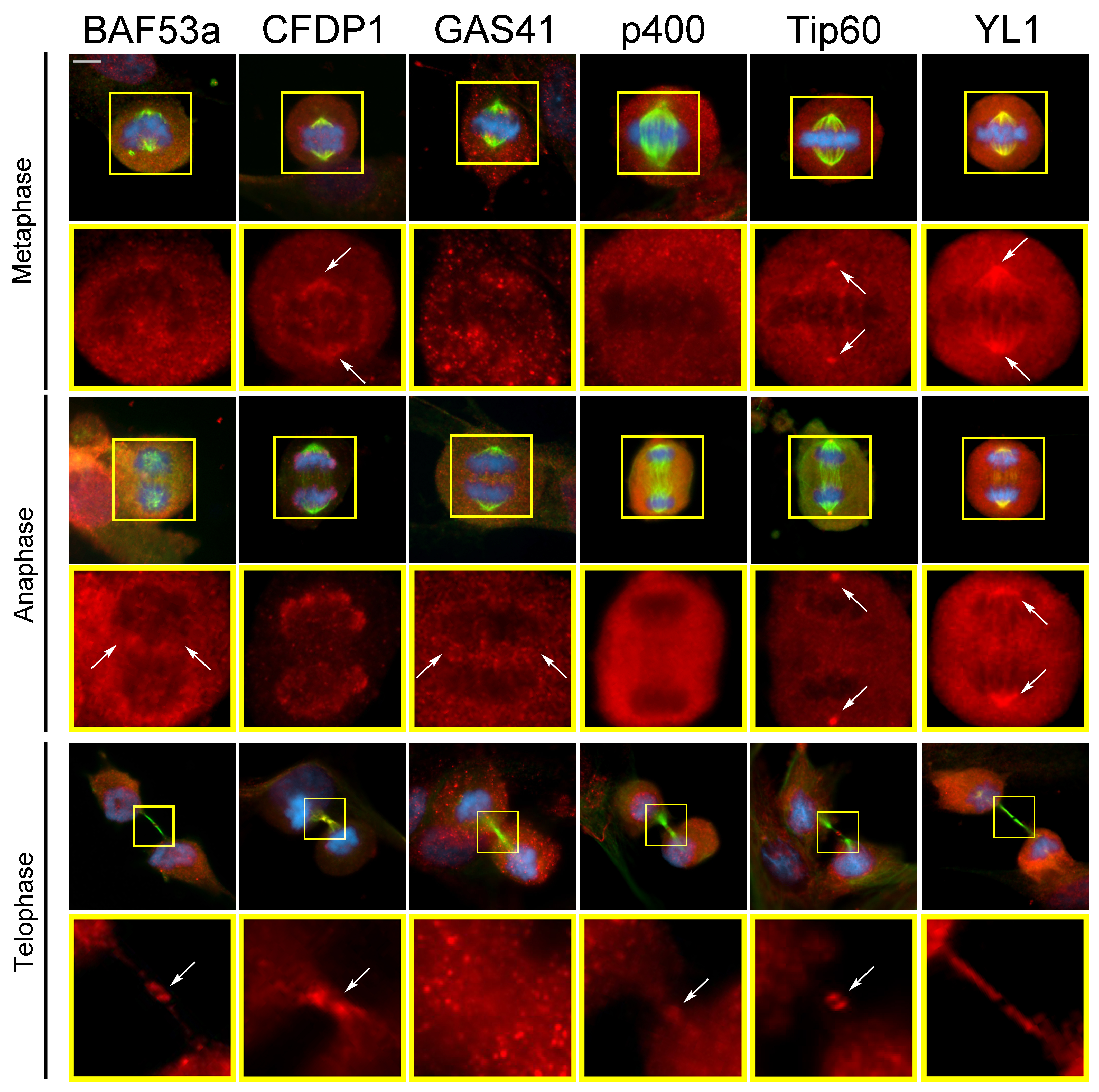

Supplement: Supplementary file 3 — Additional file 3: Figure S3. Localization of CRS to mitotic apparatus in MRC5 cells. Fixed MRC5 cells stained with DAPI (blue), antibody against the CRS of interest (red) and anti-α-tubulin (green). CFDP1 and YL1 localized to the spindle, while Tip60 the centrosomes in metaphase and anaphase; BAF53a and GAS41 localized to the central spindle in anaphase. All the subunits, with the exception of GAS41, were found at the midbody. Scale bar = 10 μm. [file 12915_2022_1365_MOESM3_ESM.jpg]

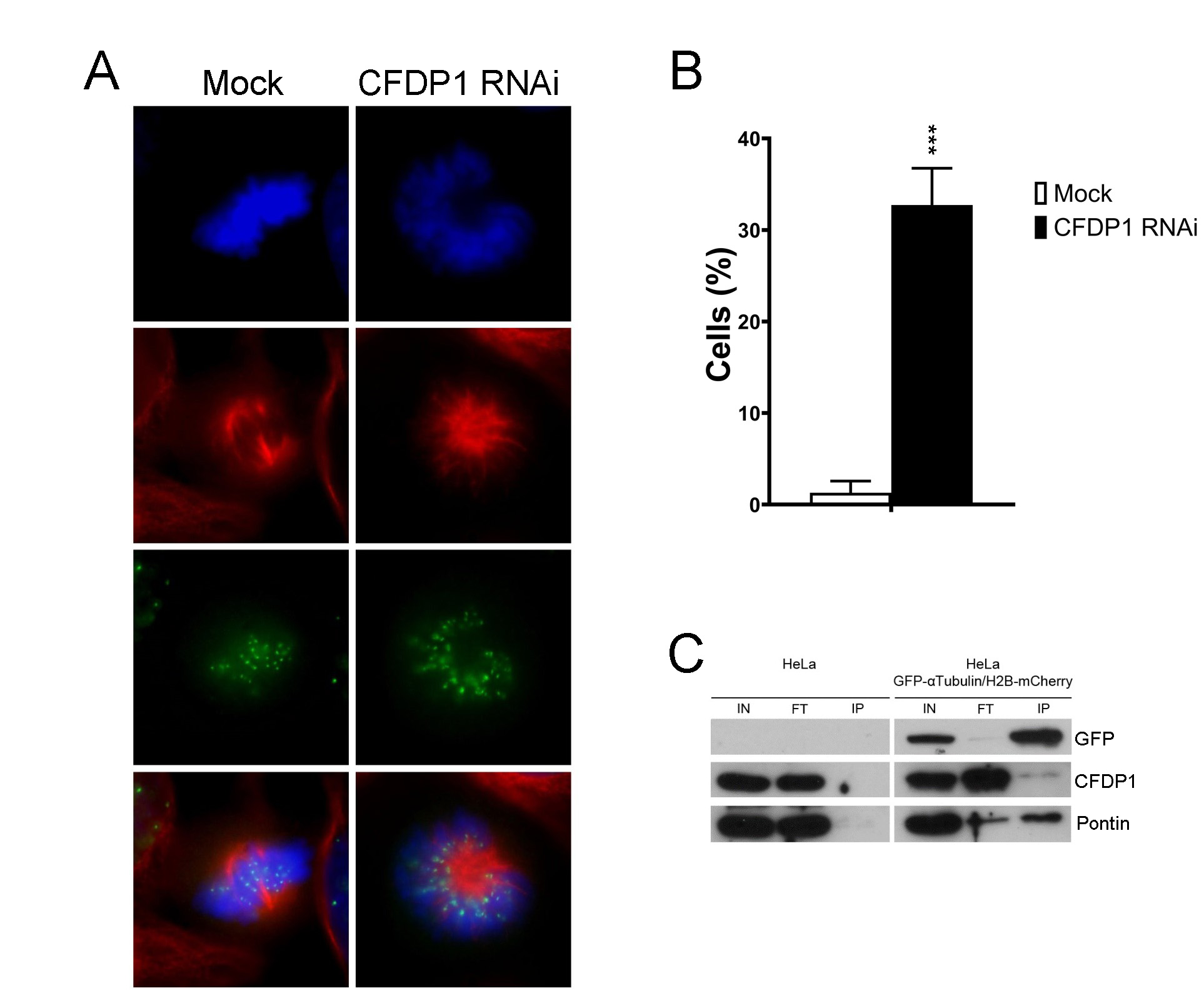

Supplement: Supplementary file 4 — Additional file 4: Figure S4. Depletion of CFDP1 in HeLa cells and interactions with a-tubulin. A) Fixed HeLa cells stained with DAPI (blue), anti-a-tubulin (red) CREST antibody (green). Scale bar = 10 μm. B) Histograms showing the % of HeLa cells with monopolar spindle after CFDP1 depletion (black histogram), compared to the control (white histogram); Three independent experiments were performed. The quantitative analysis of defects scored in RNAi-treated and control cells is based on the following numbers: at least 100 prometaphases and metaphases. Experimental group were compared with mock by Fisher's exact test. ***=P < 0.0005 C) Immunoprecipitation of EGFP::α-tubulin from asynchronized whole cell extracts. IP sample from stable EGFP::α-tubulin/mCherry::H2B HeLa cells immunoprecipitated with GFP-Trap were compared to negative controls (sample from HeLa cells). CFDP1 and Pontin (positive control) were found in the IP from EGFP::α-Tubulin/mCherry::H2B, but not in HeLa cells samples. Three independent IP experiments were performed. IN = input, FT= Flow Through, IP = immunoprecipitation. [file 12915_2022_1365_MOESM4_ESM.jpg]

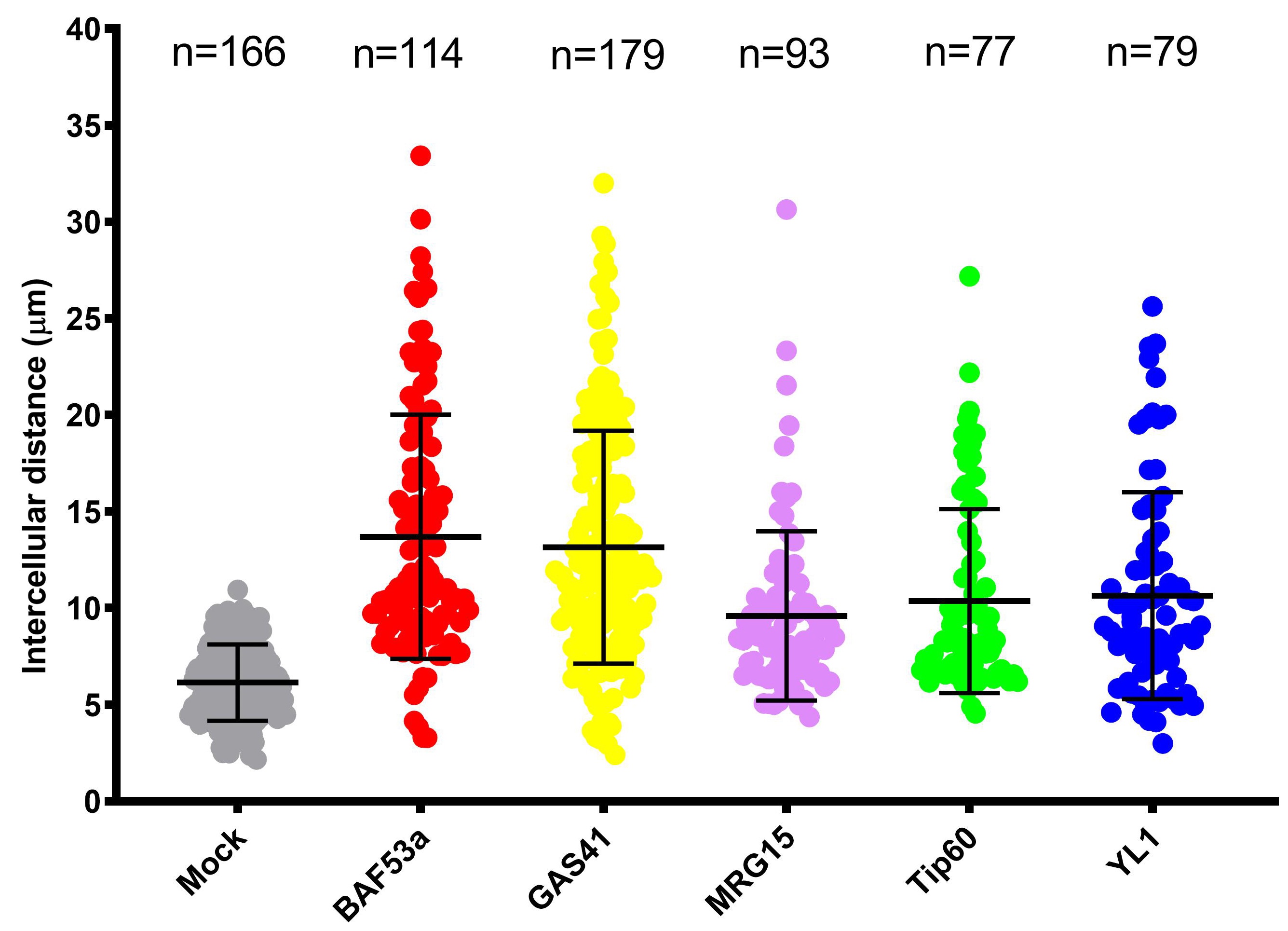

Supplement: Supplementary file 5 — Additional file 5: Figure S5. Intercellular distance. Depletion of CRS increased the intercellular distance during cytokinesis. Colors are referred to Fig. 1B. Three independent experiments were performed; statistical significance was verified by T- test. [file 12915_2022_1365_MOESM5_ESM.jpg]

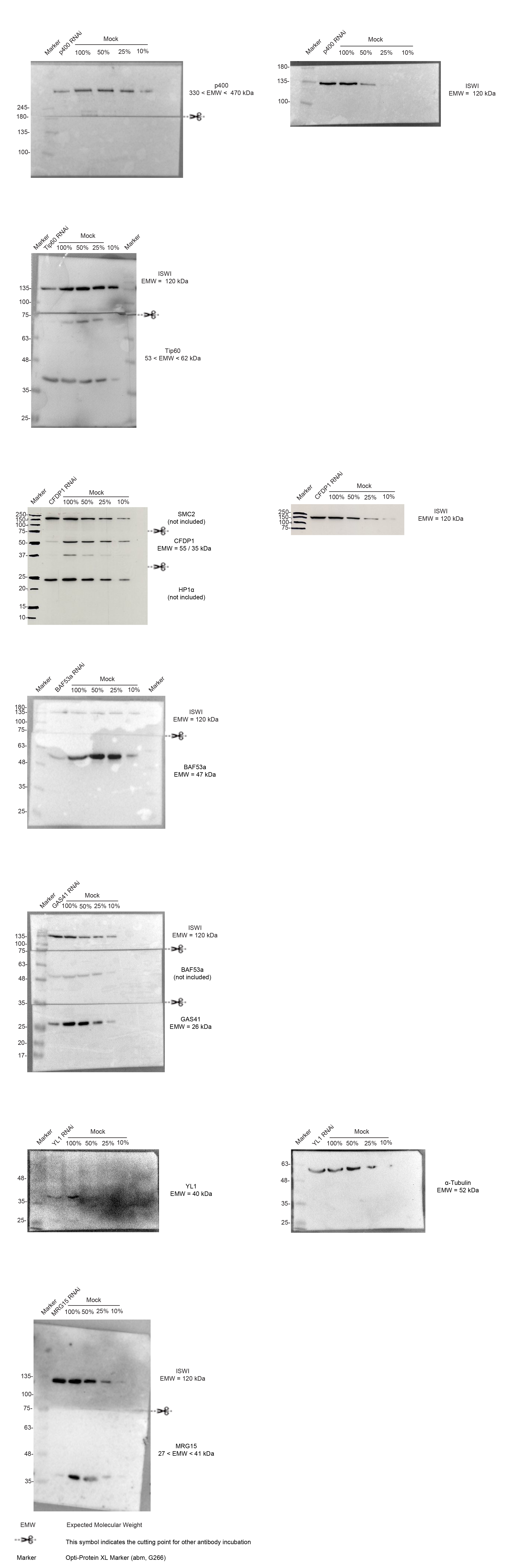

Supplement: Supplementary file 11 — Additional file 11. Uncropped blots Fig. 3A. [file 12915_2022_1365_MOESM11_ESM.jpg]

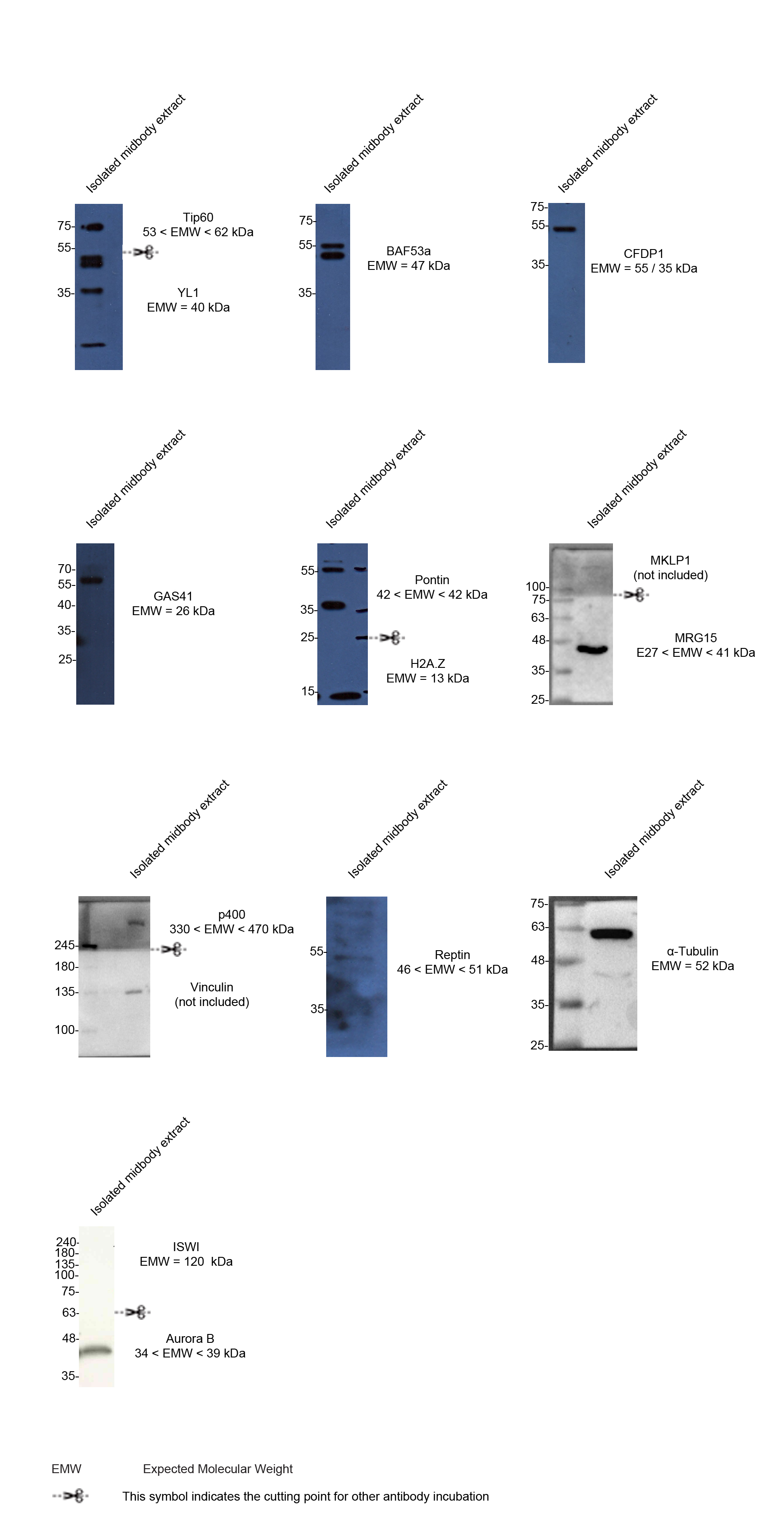

Supplement: Supplementary file 12 — Additional file 12. Uncropped gels/blots Fig. 4B. [file 12915_2022_1365_MOESM12_ESM.jpg]

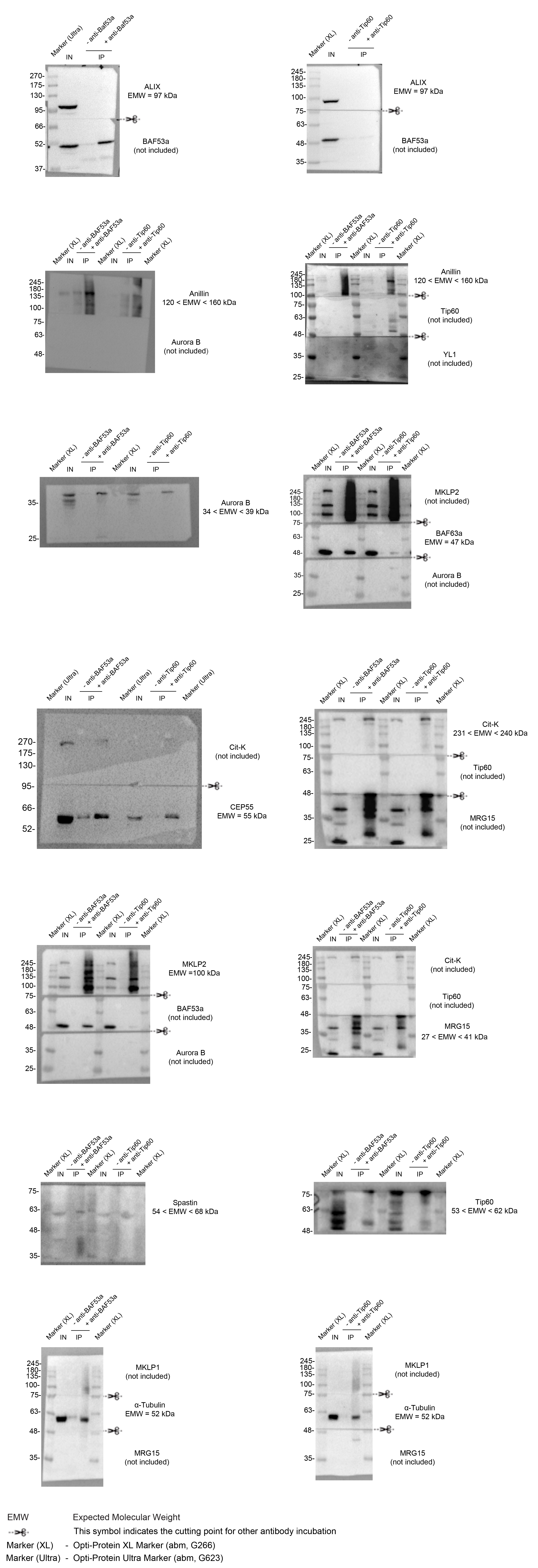

Supplement: Supplementary file 13 — Additional file 13. Uncropped gels/blots Fig. 6A. [file 12915_2022_1365_MOESM13_ESM.jpg]

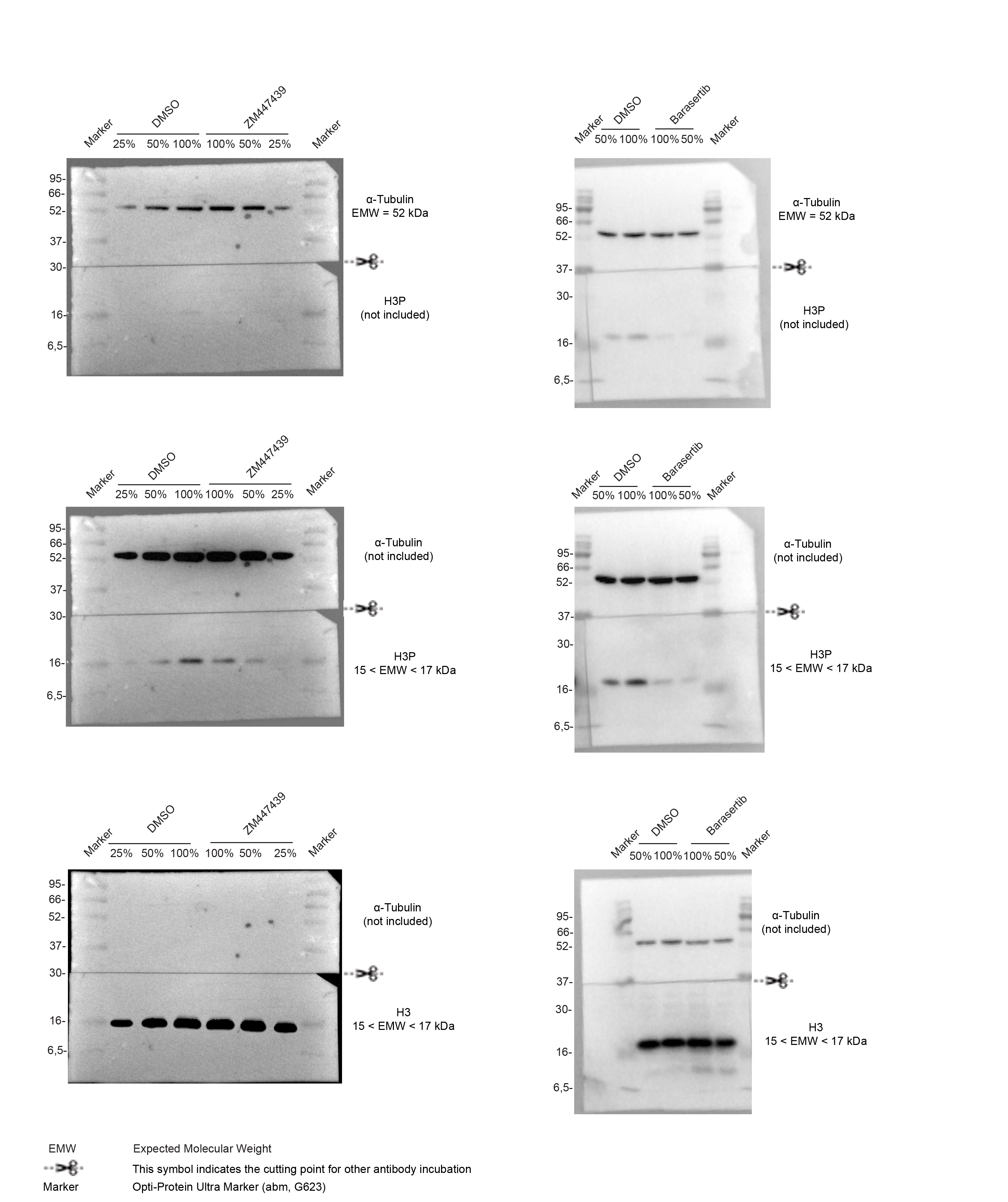

Supplement: Supplementary file 14 — Additional file 14. Uncropped gels/blots Fig. 7C, E. [file 12915_2022_1365_MOESM14_ESM.jpg]

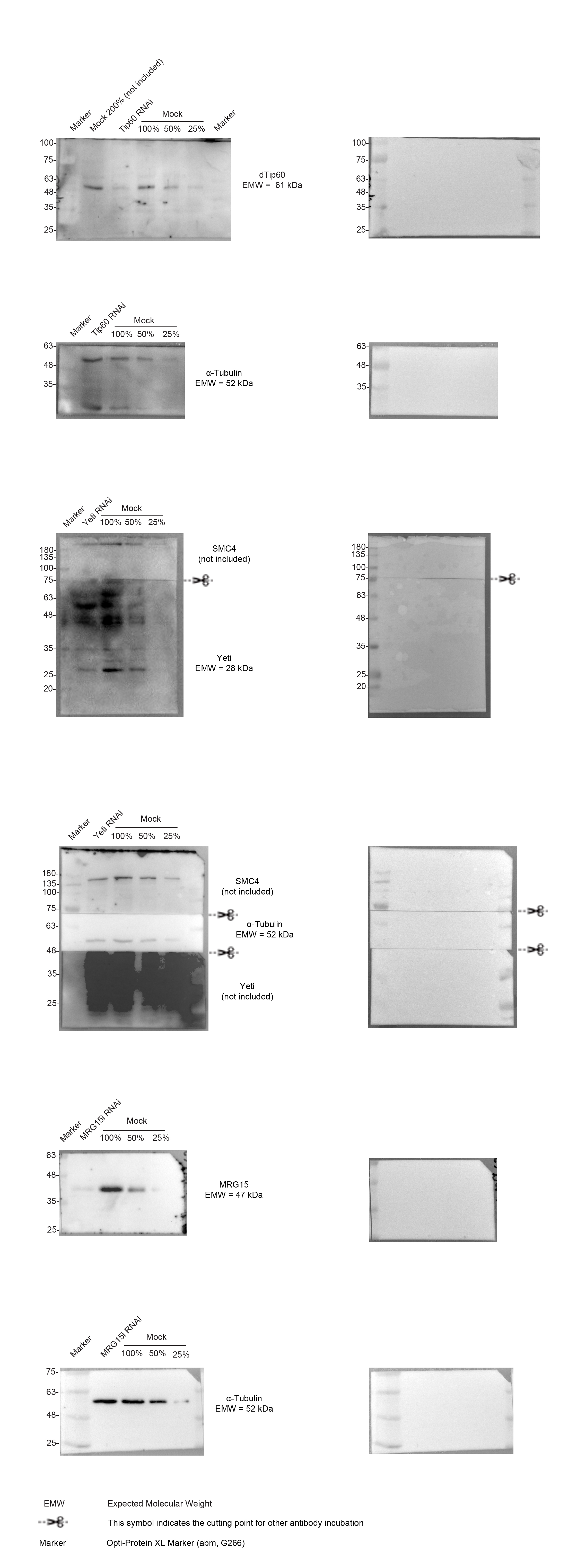

Supplement: Supplementary file 15 — Additional file 15. Uncropped gels/blots Fig. 11H. [file 12915_2022_1365_MOESM15_ESM.jpg]
