# Supplementary material for: Evolutionary conserved relocation of chromatin remodeling complexes to the mitotic apparatus
Source: BMC Biol. 2022 Aug 3;20:172. doi: 10.1186/s12915-022-01365-5 (PMC9351137; doi:10.1186/s12915-022-01365-5)
Supplement: Supplementary file 6 — Additional file 6: Table S1. List of siRNAs (Human). [file 12915_2022_1365_MOESM6_ESM.docx]

**Additional file 6: Table S1**. siRNA (Human)

| **Target** | **Sequence (5'→3')** | **Target Sequence** | **Type** | **Catalog Number** |
| --- | --- | --- | --- | --- |
| BAF53a | Strand A Sense: GCAGAGAACUCUUCCAAGAtt |  | Pool of 3 | sc-60239 (Santa Cruz) |
|  | Strand B Sense: GUAUGCGGUUGAAAUUGAUtt |  |  |  |
|  | Strand C Sense: CCAGUGGAUUGGUAGAACUtt |  |  |  |
| CFDP1 |  | CACAGAAAACCCAAGGGAAAAAAAGAAAGGCCCAGAGCATTCCAGCCAGGAAGAGAAGACAAGGTGGCCTCTCATTAGAAGAAGAGGAAGAGGAGGATGCCAATTCAGAATCTGAGGGAAGCAGTAGTGAGGAGGAAGATGACGCTGCAGAGCAGGAAAAAGGCATTGGATCAGAGGATGCCAGGAAAAAGAAGGAGGACGAACTCTGGGCCAGCTTCCTCAATGATGTGGGACCAAAATCAAAAGTGCCCCCAAGTACACAAGTTAAGAAAGGAGAGGAGACTGAAGAGACAAGTTCAAGTAAATTGTTGGTAAAAGCAGAAGAGCTAGAGAAACCTAAAGAAACAGAAAAAGTTAAAATCACCAAGGTGTTTGATTTTGCTGGTGAAGAAGTAAGGGTAACTAAGGAAGTGGATGCTACATCTAAAGAGGCCAAATCCTTCTTCA | Pool | EHU132391 (Sigma) |
| GAS41 | Strand A Sense: CCGUGGGACAAUAUGUUCAtt |  | Pool of 3 | sc-77331 (Santa Cruz) |
|  | Strand B Sense: GCUAAAGCUGUUUCAAUCAtt |  |  |  |
|  | Strand C Sense: GGUAGUAAGCUAAACUGAAtt |  |  |  |
| MRG15 (MORFL) |  | ACCGTGGCTTGTTGATGACTGGGACTTAATTACCAGGCAAAAACAGCTCTTTTATCTTCCTGCCAAGAAGAATGTGGATTCCATTCTTGAGGATTATGCAAATTACAAGAAATCTCGTGGAAACACAGATAATAAGGAGTATGCGGTTAATGAAGTTGTGGCAGGGATAAAAGAATACTTCAACGTAATGTTGGGTACCCAGCTACTCTATAAATTTGAGAGACCACAGTATGCTGAAATTCTTGCAGATCATCCCGATGCACCCATGTCCCAGGTGTATGGAGCGCCACATCTCCTGAGATTATTTGTACGAATTGGAGCAATGTTGGCTTATACACCTCTGGATGAGAAGAGCCTTGCTTTATTACTCAATTATCTTCACGATTTCCTAAAGTACCTGGCAAAGAATTCTGCAACTTTGTTCAGTGCCAGCGATTATGAAGTGGCTCCTCCTGAGT | Pool | EHU127521 (Sigma) |
| P400 |  | ACAGGGTGACACAGCCATTTATTTTGAGGAGAACTAAGAGAGATGTGGAAAAGCAACTAACAAAGAAATATGAGCATGTTTTGAAGTGTCGCCTTTCTAACCGACAAAAAGCCTTATACGAGGACGTTATCCTGCAACCTGGCACTCAGGAGGCCTTGAAGAGCGGGCACTTTGTCAACGTCCTGAGCATCCTTGTGCGGCTGCAGCGCATCTGCAACCACCCTGGGCTCGTCGAGCCCCGGCACCCAGGCTCTTCCTACGTGGCGGGGCCACTGGAGTATCCGTCCGCATCTCTAATCCTGAAGGCACTGGAGA | Pool | EHU099591 (Sigma) |
| Tip60 (KAT5) |  | GTCACCCGGATGAAGAACATTGAGTGCATTGAGCTGGGCCGGCACCGCCTCAAGCCGTGGTACTTCTCCCCGTACCCACAGGAACTCACCACATTGCCTGTCCTCTACCTGTGCGAGTTCTGCCTCAAGTACGGCCGTAGTCTCAAGTGTCTTCAGCGTCATTTGACCAAGTGTGACCTACGACATCCTCCAGGCAATGAGATTTACCGCAAGGGCACCATCTCCTTCTTTGAGATTGATGGACGTAAGAACAAGAGTTATTCCCAGAACCTGTGTCTTTTGGCCAAGTGTTTCCTTGACCATAAGACACTGTACTATGACACAGACCCTTTCCTCTTCTACGTCATGACAGAGTATGACTGTAAGGGCTTCCACATCGTGGGCTACTTCTCCAAGGAGAAAGAATCAACGGAAGACTACAATGTGGCCTGCATCCTAAC | Pool | ImmunoReagents |
| YL1 (VPS72) |  | TGATGAAGGGGATGAACCATCCAGTGATGGAGAAGCAGAAGAGCCAAGAAGGAAGCGCCGAGTAGTCACCAAGGCCTATAAGGAACCTCTCAAGAGCTTAAGGCCTCGAAAGGTCAACACCCCGGCTGGTAGCTCTCAGAAGGCGCGAGAAGAGAAGGCACTACTGCCATTAGAACTACAAGATGACGGCTCTGACAGTCGGAAGTCTATGCGTCAGTCTACAGCTGAGCATACACGACAAACGTTCCTTCGGGTACAGGAGAGGCAGGGCCAGTCAAGACGGCGAAAGGGGCCCCACTGTGAGCGGCCACTAACCCAGGAGGAACTGCTCCGGGAGGCCAAGATCACAGAAGAGCTTAATTTACGGTCACTGGAGACATATGAGCGGCTCGAGGCTGATAAAAAGAAGCAGGTTCATAAGAAGCGG | Pool | EHU090771 (Sigma) |
