# Supplementary material for: Evolutionary conserved relocation of chromatin remodeling complexes to the mitotic apparatus
Source: BMC Biol. 2022 Aug 3;20:172. doi: 10.1186/s12915-022-01365-5 (PMC9351137; doi:10.1186/s12915-022-01365-5)
Supplement: Supplementary file 7 — Additional file 7: Table S2. Oligos for siRNAs synthesis (Drosophila S2 cells). [file 12915_2022_1365_MOESM7_ESM.docx]

**Additional file 7: Table S2.** Oligos for siRNA synthesis (*Drosophila* S2 cells)

| **Target** | **Name** | **Sequence** | **Length** | **PCR fragment size (bp)** |
| --- | --- | --- | --- | --- |
| Mrg15 | Mrg15_For | GAATTAATACGACTCACTATAGGGAGAC TACGCAGCTAAGGTGGAGGT | 48 | 525 |
|  | Mrg15_Rev | GAATTAATACGACTCACTATAGGGAGAC CTGTGCATTTCGCACGTACT | 48 |  |
| Tip60 | Tip60_ For | GAATTAATACGACTCACTATAGGGAGACCCCTTCAGAAACGCATCAAT | 48 | 511 |
|  | Tip60_ Rev | GAATTAATACGACTCACTATAGGGAGACACCTTGTTTTTGCGTCCATC | 48 |  |
| Yeti | Yeti_For | GAATTAATACGACTCACTATAGGGAGACATCGCGGTCGGATGCTTTAT | 48 | 554 |
|  | Yeti_Rev | GAATTAATACGACTCACTATAGGGAGACCCAAATACCCGTCCTTGCCT | 48 |  |
