# Supplementary material for: Evolutionary conserved relocation of chromatin remodeling complexes to the mitotic apparatus
Source: BMC Biol. 2022 Aug 3;20:172. doi: 10.1186/s12915-022-01365-5 (PMC9351137; doi:10.1186/s12915-022-01365-5)
Supplement: Supplementary file 8 — Additional file 8: Table S3. List of primary antibodies. [file 12915_2022_1365_MOESM8_ESM.docx]

**Additional file 8: Table S3.** Primary antibodies

| **Epitope** | **Reacts** | **Host** | **MW (kDa)** | **WB** | **IF** | **Description** | **Supplier** | **Catalog Number** |
| --- | --- | --- | --- | --- | --- | --- | --- | --- |
| Alix | Human | Ms | 96 |  | 1:100 | Alix Antibody (3A9) | SantaCruz | sc-53538 |
| Anillin | Human | Rb | 130 |  | 1:500 | Anillin Antibody | Bethyl | A301-406A |
| AuroraB | Human | Rb | 39 | 1:2000 | 1:1000 | Anti-Aurora B antibody | Abcam | ab3254 |
| AuroraB | Human | Ms | 39 | 1:1000 | 1:100 | AIM-1 | BD Transduction Laboratories™ | 611083 |
| CEP55 | Human | Ms | 55 | 1:1000 | 1:100 | Anticorpo CEP55 (B-8) | SantaCruz | sc-374051 |
| BAF53a | Human | Rb | 53 | 1:1000 | 1:50 | Anti-BAF53a Antibody ChIP Grade | Abcam | ab3882 |
| BAF53a | Human | Ms | 53 | 1:1000 | 1:50 | BAF53a (E-3) | SantaCruz | sc-137062 |
| CFDP1 | Human | Ms | 50 -37 | 1:1000 | 1:100 | Monoclonal anti-CFDP1 producted in mouse, clone 5B7 | Sigma | WH0010428M4 |
| Cit-K | Human | Ms | 240 | 1:2000 | 1:250 | Purified Mouse Anti-CRICK | BD Transduction Laboratories™ | 611376 |
| GAS41 | Human | Ms | 26 | 1:500 | 1:50 | Anti-GAS41 antibody | Abcam | ab167495 |
| H2Az | Human | Rb | 17 | 1:500 | 1:100 | Anti-Histone H2A.Z antibody - ChIP Grade | Abcam | ab4174 |
| H3 | Drosophila/Human | Rb | 16 | 1:1000 |  | Anti-Histone H3 Antibody | Millipore | 06-755 |
| H3S10phospho | Human | Rb | 18 | 1:500 | 1:100 |  | A. Losada |  |
| ISWI | Human | Rb | 150 | 1 µg/ml | 1 µg/ml |  | T. Hirano |  |
| MKLP1 | Human | Ms | 110 |  | 1:100 | Anti-MKLP1 Antibody | Abcam | ab23956 |
| MKLP2 | Human | Rb | 100 | 1:1000 | 1:250 |  | Thomas Mayer |  |
| MRG15 | Mouse/Human | Rb | 42 | 1:1000 | 1:200 | MORF4L1 | Kaoru Tominaga |  |
| MRG15 | Drosophila | Rb |  | 1:1000 | 1:1000 |  | G. Bosco |  |
| p400 | Human | Rb | 400 | 1:1000 | 1:400 | Anti-p400 antibody – ChIP Grade | Abcam | ab70301 |
| Spastin | Human | Ms | 54/67 |  | 1:100 | Spastin Antibody (Sp 3G11/1) | SantaCruz | sc-53443 |
| Tip60 | Drosophila | Gp | 60 | 1:500 | 1:100 |  | AE. Ehrenhofer-Murray |  |
| Tip60 | Human | Ms | 60 | 1:1000 | 1:100 | Anti Tip60 (C-7) | SantaCruz | sc-166323 |
| Tip60 | Human | Rb | 60 | 1:250 | 1:100 | Anti-KAT5 / Tip60 antibody | Abcam |  |
| SRCAP | Human | Rb | 350 |  | 1:100 | SRCAP (T-15) | SantaCruz | sc-133312 |
| αTubulin | Drosophila/Human | Ms | 50 | 1:20000 | 1:500 | Monoclonal DM1A | Sigma-Aldrich | T9026 |
| αTubulin | Drosophila/Human | Rb | 50 |  | 1:2000 | Anti-alpha Tubulin antibody - Microtubule Marker | Abcam | ab18251 |
| βActin | Drosophila/Human | Ms | 42 | 1:10000 |  | Beta-Actin Antibody - mouse monoclonal antibody | Abgent | AM102b |
