# Supplementary material for: Evolutionary conserved relocation of chromatin remodeling complexes to the mitotic apparatus
Source: BMC Biol. 2022 Aug 3;20:172. doi: 10.1186/s12915-022-01365-5 (PMC9351137; doi:10.1186/s12915-022-01365-5)
Supplement: Supplementary file 9 — Additional file 9: Table S4. List of secondary antibodies. [file 12915_2022_1365_MOESM9_ESM.docx]

**Additional file 9: Table S4**. Secondary antibodies

| **React** | **Conjugation** | **Host** | **Description** | **Diluition** | **Supplier** | **Catalog Number** |
| --- | --- | --- | --- | --- | --- | --- |
| Guinea Pig | Cy3 | Goat | Cy™3 AffiniPure Goat Anti-Guinea Pig IgG (H+L) | 1:200 | Jackson ImmunoResearch | 106-165-003 |
| Mouse | 488 | Chicken | Chicken anti-Mouse IgG (H+L) Cross-Adsorbed Secondary Antibody, Alexa Fluor 488 | 1:200 | Invitrogen | A21200 |
| Mouse | 555 | Goat | Goat anti-mouse IgG Secondary Antibody Alexa Fluor 555 | 1:200 | Invitrogen | A21422 |
| Mouse | HRP | Goat | goat anti-rabbit IgG-HRP | 1:1000 | Santa Cruz | sc-2005 |
| Rabbit | 488 | Chicken | Chicken anti-Rabbit IgG (H+L) Cross-Adsorbed Secondary Antibody, Alexa Fluor 488 | 1:200 | Invitrogen | A21441 |
| Rabbit | 555 | Goat | F(ab')2-Goat anti-Rabbit IgG (H+L) Cross-Adsorbed Secondary Antibody, Alexa Fluor 555 | 1:200 | Invitrogen | A21430 |
| Rabbit | HRP | Goat | Goat anti rabbit IgG (H&L) conjugate HRP (1 mg) | 1:5000 | ImmunoReagents | GTXRB-003-DHRPX |
